# β‐Turn Mimicking Crosslinking Provides Hyperstability and Fast Folding Kinetics for Short Collagen Triple Helices

**DOI:** 10.1002/cbic.202400834

**Published:** 2025-03-19

**Authors:** Pengfei Jin, Diane N. Rafizadeh, Huanyi Zhao, David M. Chenoweth

**Affiliations:** ^1^ Department of Chemistry University of Pennsylvania Philadelphia Pennsylvania 19104 United States

**Keywords:** collagen mimetic peptides, peptide crosslinking, protein folding kinetics, stable collagen triple helices, collagen heterotrimer

## Abstract

Creating stabilized peptide mimics of the collagen triple helix is challenging, especially for collagen heterotrimers. Interstrand sidechain crosslinking offers a useful approach, though this strategy can suffer from destabilizing structural perturbations, sequence limitations and synthetic complexity. Herein, we show that the geometry of hydrogen bonding in the collagen triple helix is compatible with installation of terminal β‐turn‐mimicking linkers at the N‐terminal and C‐terminal ends of the triple helix. These double‐turn‐containing collagen peptide mimics fold into highly stable, intramolecular triple helical structures, providing access to profoundly miniaturized triple helix mimics. Intramolecular triple helix formation exhibits significantly accelerated folding kinetics. Comprehensive kinetic analysis reveals that the rate‐limiting step of folding is distinct at low and high temperatures, affording unique insight into the mechanism.

## Graphical abstract

Collagen triple helices and β‐turns are compatible chemical motifs to fuse based on their identical H‐bond geometry. The resulting collagen triple helices are intramolecularly folded, exhibiting high stability and fast folding kinetics. Besides serving as a scaffold structure for designing short, stable, heterotrimeric collagen triple helices, the bis β‐turn collagen peptides were used to deconvolute a two‐step collagen folding mechanism as a kinetic model.

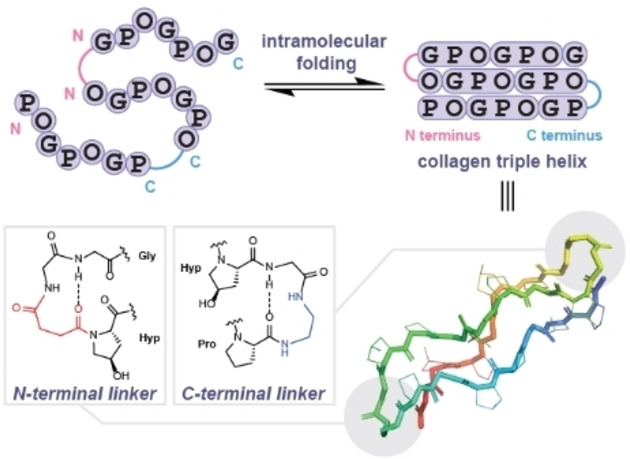

## Introduction

Collagen is the most abundant protein in the human body and is widely recognized for its pivotal role in forming the structural framework of various tissues.^[1]^ To study the biology of collagen and prepare collagen‐like functional materials, collagen mimetic peptides (CMPs) are commonly used as a simplified model system and as building blocks.^[2]^ CMPs are short peptides that can take on the triple helical structure of natural collagen and consist of the collagen characteristic triplet repeat sequence (Gly‐X_aa_‐Y_aa_). Proline (Pro, P) and hydroxyproline (Hyp, O) have the greatest propensity in natural collagen for the X_aa_ and Y_aa_ positions,[^3^, ^4^] respectively, as they provide the highest stability for the collagen triple helix;^[5]^ thus, the (Gly‐Pro‐Hyp) triplet is commonly used in designing CMP sequences. Typically, a sequence of 7−10 triplets in a CMP is sufficient to form a stable trimeric collagen triple helix, and the sequence is variable depending on the demanding function.^[6]^ As natural collagen mimics can be facilely prepared and modified by solid‐phase peptide synthesis (SPPS), CMPs are widely used in collagen‐related studies, including those investigating collagen self‐assembly, collagen‐protein interactions, wound healing, tissue engineering, and drug delivery.[^2^, ^7^, ^8^]

In this work, we introduce a new strategy for modifying CMPs by installing terminal β‐turn‐mimicking linkers connecting three collagen peptide strands to achieve intramolecular triple helical self‐assembly with high triple helix stability. Crosslinking is one of the most prevalent modifications of CMPs for several reasons as follows: (1) *Stabilization of the triple helix and miniaturization of collagen mimics*: Connecting the three peptide chains covalently would switch the peptide folding process from intermolecular to intramolecular, substantially reducing the entropic penalty and thereby stabilizing the collagen triple helix. Additionally, the enhanced stability allows the preparation of shorter stable triple helices, as in most collagen‐protein interactions, the interface between the triple helix and the protein is relatively small, typically spanning 2−4 triplets.[^9^, ^10^, ^11^, ^12^, ^13^, ^14^, ^15^, ^16^, ^17^] Therefore, functional CMPs could be designed to be shorter in length with facilitated synthesis as an alternative to common CMPs with 7−10 triplets in length. (2) *Construction of heterotrimeric collagen mimics*: Many human collagen types have heterotrimeric structures.[^1^, ^6^] Thus, the ability to make heterotrimeric collagen mimics is essential for collagen‐related studies. However, heterotrimeric CMPs have historically been difficult to achieve synthetically. Collagen peptide trimerization is relatively nonspecific, and the selective formation of heterotrimers is very challenging, especially when specific functional sequences are required to be incorporated into the peptide sequences.[^18^, ^19^, ^20^, ^21^] Crosslinking provides a direct way to ensure heterotrimer formation because it covalently tethers three different strands together.

Numerous studies have focused on crosslinking collagen mimetic peptides employing diverse chemistries. Trifunctional templating linkers are mostly used to connect peptides at their termini, and alternatively, multiple bridging linkers can crosslink three peptide strands together at either the termini or the middle of the peptide. Diverse chemistry has been developed in the crosslinking moiety, in which amide bond‐forming crosslinking is predominantly used[^22^, ^23^, ^24^, ^25^, ^26^, ^27^, ^28^, ^29^, ^30^, ^31^, ^32^, ^33^, ^34^, ^35^, ^36^, ^37^, ^38^, ^39^, ^40^, ^41^, ^42^, ^43^] alongside other methods including disulfide bonding,[^44^, ^45^, ^46^, ^47^, ^48^, ^49^, ^50^, ^51^] oxime ligation,^[52]^ metal coordination complexes,[^53^, ^54^] and borate ester formation.^[55]^

Like other proteins, collagen has a delicate folded structure that is sensitive to modification or conformational disruptions. The known crosslinking methods described above result in varied structural stability due to differences in the crosslinking moiety, even with the same molecular topology. The structure of the crosslinking linker could thus have a critical effect on collagen stability, which has not been well studied. Improper geometry can disrupt the triple helix and destabilize the structure if the linker is too short or the proper bond geometry is not satisfied.

**Figure 1:**
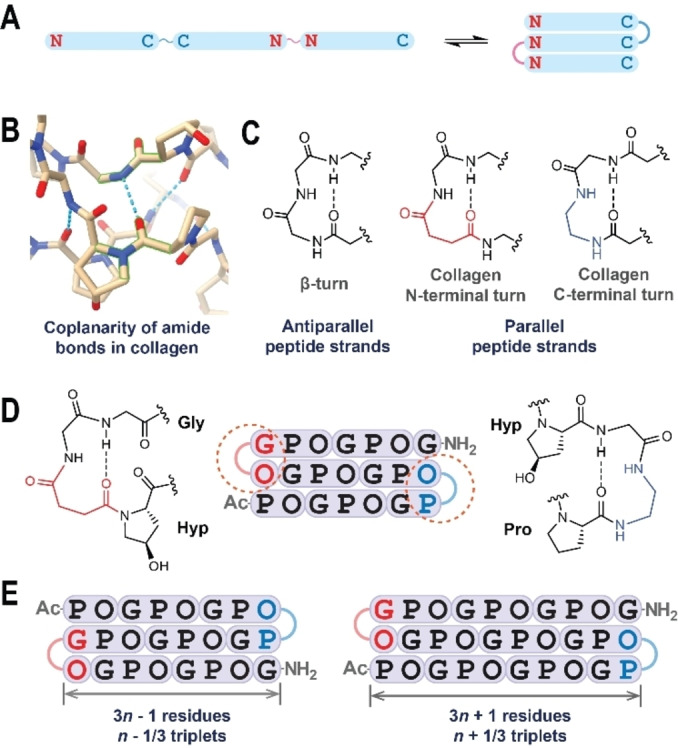
Design of the bis β‐turn CMP terminal turn structures. (A) The primary structure of a bis β‐turn CMP comprises three peptide segments and two linkers, in which the reversed peptide backbone of the middle segments allows the molecule to fold into a unimolecular triple helix. The N‐ and C‐termini of the peptide segments are labeled with “N” and “C,” respectively. (B) The local structure of collagen (PDB: 3B0S) presents an interstrand hydrogen bond. The two involved amide bond planes (green contour labeled) are approximately coplanar. (C) Chemical structures of a β‐turn as compared to the proposed collagen‐compatible turn structures at the N‐terminus and C‐terminus. (D) Example of a bis β‐turn CMP sequence and its terminal turn structures; loop‐connected residues are highlighted in red or blue. (E) The two types of symmetries of bis β‐turn CMPs. The N‐ and C‐terminus are capped with acetyl and amide groups, respectively, and the triple helix length is characterized by the number of triplets, *n*.

To address this problem, we designed and synthesized bis β‐turn CMPs. Our approach involves the incorporation of a linker at each terminus of the triple helix, thereby creating a linear molecular structure comprised of three segments with alternating peptide backbone direction (Figure 1A). To avoid disruption of the triple helical structure, minimal linkers are used to mimic a β‐turn structure. Through inspection of the collagen triple helix (PDB: 3B0S),^[56]^ we observed that each pair of amide bonds that form the intermolecular hydrogen bonds are nearly aligned in the same plane. This geometry closely resembles a β‐turn structure, where the two amide bonds involved in hydrogen bonding at the turn are also coplanar (Figure 1B). Designing biomimetic turn structures at the ends of the collagen triple helix with a similar length to the β‐turn could provide interstrand connectivity without changing the native collagen hydrogen bond conformation, ensuring inherent compatibility with the collagen triple helix structure.

β‐turns typically link two polypeptide chains in an anti‐parallel fashion, whereas the strands in collagen are parallel. To accommodate this difference, bifunctional moieties are required to connect the peptide termini: at the *N*‐terminus, we incorporate a dicarboxylic acid linker, and at the *C*‐terminus, we utilize a diamine linker. These bifunctional linkers have been proven compatible in reversing the peptide backbone direction in parallel beta‐sheet mimicking foldamers when the linker length and substitution are properly designed.[^57^, ^58^, ^59^] In our design, we choose succinic acid as the *N*‐terminal linker and ethylenediamine as the *C*‐terminal linker. Combining these two turns with a glycine residue and ensuring that the turns have the same backbone length as β‐turns allows for compatibility with the triple helix hydrogen bonds of collagen (Figure 1C). In collagen triple helices, the hydrogen bonds are formed between the glycine nitrogen and proline carbonyl, necessitating the β‐turn mimicking linker to connect glycine and hydroxyproline at the N‐terminus in addition to connecting hydroxyproline and proline at the C‐terminus (Figure 1D). Using this specific connectivity, we can derive the whole sequence of a bis β‐turn CMP from a specific length. The three collagen mimicking segments have equal length, but the sequences are permutated to fit the staggered self‐assembly symmetry of the natural collagen triple helix, which has a one amino acid offset between the strands. Dictated by the Gly‐Hyp linkage at the N‐terminus and the Hyp‐Pro linkage at the C‐terminus, the turns at both termini could take two distinct relevant positions, resulting in peptides with 3*n*−1 or 3*n*+1 residues in length in the triple‐helical region (Figure 1E), whereas the configuration with 3*n* in helix length does not give a single molecule due to the formation of a loop (Figure S1).

## Results and Discussion

**Figure 2:**
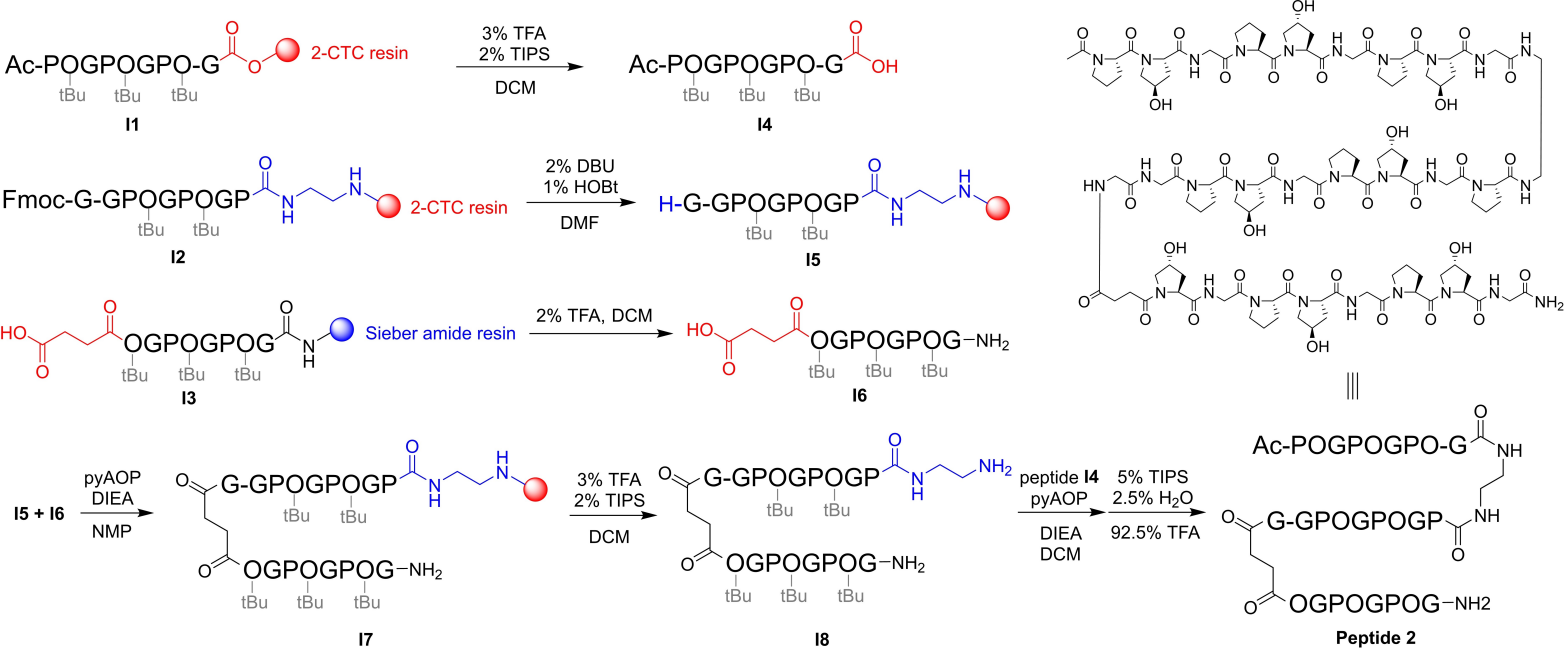
Schematic of the synthetic route to a bis β‐turn CMP.

The synthesis of bis β‐turn CMPs was performed by solid‐phase peptide synthesis. Taking peptide **2** as an example (Figure 2), three peptide fragments (**I4**, **I5**, **I6**) were first synthesized separately on resin via standard SPPS followed by two further coupling reactions to connect the three strands using common peptide synthesis coupling reagents. This linkage of the protected individual strands was performed on resin to prepare intermediate **I7** and then in the liquid phase to form **I8**. The peptide final product, Peptide **2**, was obtained after side chain deprotection.

**Figure 3:**
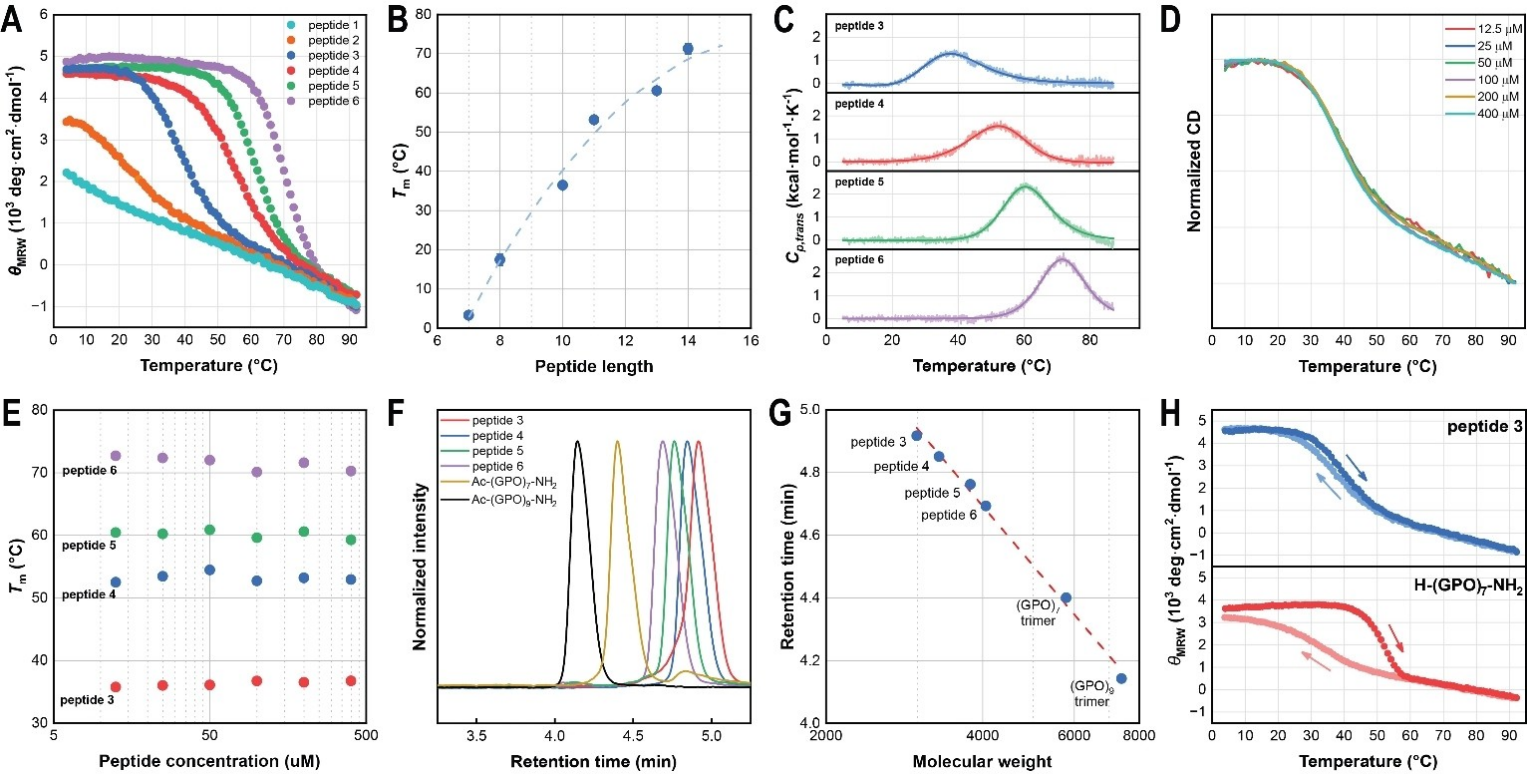
Self‐assembly characterization of bis β‐turn CMPs by CD, SEC, and DSC. (A) CD thermal denaturation curves of bis β‐turn CMPs at 200 μM peptide in PBS, measured at 225 nm. (B) Plot of peptide *T_m_* versus length of the triple helical region. (C) CD thermal denaturation curve of peptide **3** at varied peptide concentrations in PBS. (D) *T_m_* values of peptides **3**–**6** measured at varied peptide concentrations in PBS. (E) SEC data of bis β‐turn CMPs and two trimeric control peptides. (F) The plot of SEC retention versus logarithm molecular weight gives a linear relationship. (G) DSC data of the peptide unfolding heat capacities of peptides **3**–**6**. Light‐colored and dark‐colored lines are the measured data and the fitted curve, respectively. (H) CD hysteresis curves of peptide **3** and H‐(GPO)_7_‐NH_2_.

To investigate the capacity of the bis β‐turn CMPs to form collagen‐like triple helices and measure stability of the folded structures, we monitored the structures of the bis β‐turn CMPs via circular dichroism (CD) at 225 nm, the collagen‐featured local maximum on CD spectra. All peptides exhibited unfolding upon heating on CD, and peptides **3**−**6** presented a clear two‐state denaturation curve (Figure 3A). The curve was fitted with a two‐state model (see Supporting Information) to determine the melting temperature (*T_m_*) at which 50 % of the peptide unfolds. For peptides **1** and **2** in PBS buffer, the peptides partially form triple helices. Due to their low *T_m_* values, the CD curves cannot give a clear two‐state transition, so a a linear extrapolation method was used to estimate the *T_m_* for each of these peptides. This method was accomplished using trimethylamine N‐oxide (TMAO) to assist collagen folding, taking advantage of the linear dependence of the *T_m_* on the TMAO concentration (Supporting Information).^[60]^ The resulting *T_m_* values are listed in Table 1. The thermal melting curves indicate that double‐turned crosslinking can dramatically stabilize the collagen triple helix. Peptide **1** has 7 residues (2 1/3 triplets) in triple helix length and can form triple helices at low temperatures in TMAO conditions. Peptide **3** is the shortest bis β‐turn CMP that forms stable triple helices at room temperature in PBS buffer, with 10 residues (3 1/3 triplets) in triple helical length. The results support the β‐turn mimicking linkers being compatible with the collagen triple helical structure. Therefore, the bis β‐turn CMP design is an effective approach to stabilize collagen triple helices and prepare small‐sized collagen mimics.

**Table 1:** Folding thermodynamics characterization data of bis β‐turn CMPs.

Peptide	Peptide sequence	Helix length (residue)	*T*_m_ (°C)	*ΔH* (kcal mol^−1^)^*b*^		*ΔS* (cal mol^−1^ K^−1^)^*b*^	
				CD	DSC	CD	DSC
**1**	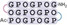	7	3^*a*^	–		–	
**2**	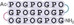	8	17^*a*^	–		–	
**3**	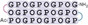	10	36	−35.7	−30.4	−115	−99.0
**4**	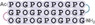	11	53	−35.5	−35.2	−109	−107
**5**	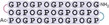	13	61	−45.1	−45.0	−137	−135
**6**	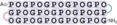	14	71	−48.4	−51.2	−141	−149

[a] *T_m_* value obtained by extrapolating peptide unfolding results measured in TMAO solutions. [b] Values of *ΔH* and *ΔS* are at *T_m_*.

The relationship between stability of the triple helix and peptide length is shown in Figure 3B; stability increases as peptide length grows. However, the relationship between *T_m_* and triple helix length is not a smooth curve but rather shows deviations, indicating that peptide length is not the only parameter that contributes to the helical stability. The *T_m_* increase between peptides **4** and **3** is more significant than the increase between peptides **5** and **4**, suggesting that the peptides with 3*n*−1 in helix length exhibit more stability than expected. Although the 3*n*−1 and 3*n*+1 configurations have the same terminal structures, differences in strand connection may lead to variations in their intrinsic stability of the triple helix. According to a previous study on aza‐glycine‐incorporated CMPs, residues that are close to the terminus have less contribution to the stability of triple helix due to their higher flexibility.^[61]^ In bis β‐turn CMPs, the middle segment is more constrained than the other two terminal segments because it is crosslinked at both termini, which is considered to contribute more to triple helix stability. In the 3n−1 configuration, the middle strand is G(POG)_n‐1_P; in the 3n+1 configuration the middle sequence is OG(POG)_n‐1_PO. We infer that this sequence difference leads to the extra stability of the 3n−1 configuration, as it has been reported that variations in terminal residues can significantly affect triple helix stability in frame‐shifted CMPs.[^62^, ^63^]

Differential scanning calorimetry (DSC) measurements were also performed for peptides **3**–**6** (Figure 3C). Changes in enthalpy and entropy from bis β‐turn CMP folding were derived by curve‐fitting both the CD and DSC data (Table 1). The thermodynamics measured by CD and DSC are largely consistent. In general, the magnitudes of the *ΔH* and *ΔS* values increase as peptide length grows. Peptides with 3*n*+1 and 3*n*+2 sequences have similar *ΔH* and *ΔS* values, whereas increasing the peptide length from 3*n*−1 to 3*n*+1 results in great increases in the *ΔH* and *ΔS* values.

Size exclusion chromatography (SEC) in addition to CD was used to verify that the bis β‐turn CMPs self‐assemble intramolecularly as proposed. We performed CD thermal denaturation for peptides **3**–**6** at different concentrations (Figure 3D, E), and all peptides demonstrated no dependence of concentration on thermal stability, which strongly supports the peptide folding being monomeric rather than multimeric. In comparison, the folding of Ac‐(GPO)_7_‐NH_2_ trimer exhibited concentration dependence (Supporting Information Figure S3). Size exclusion chromatography (SEC) was applied to determine the hydrodynamic size of the self‐assembled peptides (Figure 3F, G). Compared with the trimeric folded collagen peptides Ac‐(GPO)_7_‐NH_2_ and Ac‐(GPO)_9_‐NH_2_, peptides **3**–**6** resulted in longer retention times on SEC, consistent with their relative sizes. The plot of log(MW) versus retention time shows a clear linear relationship when we plot the data of monomeric peptides **3**–**6** and trimeric Ac‐(GPO)_7_‐NH_2_ (trimer MW=5790, *T_m_* =47 °C) and Ac‐(GPO)_9_‐NH_2_ (trimer MW=7394, *T_m_* =65 °C); hence, the retention times of the bis β‐turn CMPs on SEC are consistent with bis β‐turn CMP monomers as expected for intramolecular CMP assembly. If the bis β‐turn CMPs were intermolecularly self‐assembled into trimers or other multimeric structures, they would demonstrate relatively shorter retention times than Ac‐(GPO)_7_‐NH_2_ and Ac‐(GPO)_9_‐NH_2_. This result confirms that bis β‐turn CMPs self‐assemble intramolecularly. Additionally, we performed SEC for peptide **3** at 25 °C and 40 °C (Supporting Information). At the higher temperature, peptide **3** gave a shorter retention time and a broader peak (Figure S4B), suggesting that the unfolded peptide has a larger hydrodynamic size and a larger size distribution as a random coil, further supporting the intramolecular self‐assembly of bis β‐turn CMPs.

Since crosslinking switches collagen folding from a trimeric to a monomeric mechanism, we expected that the proximity offered by crosslinking would significantly boost the folding kinetics of the bis β‐turn CMPs. We collected CD hysteresis data for peptide **3** and compared it with that of H‐(GPO)_7_‐NH_2_ (*T_m_* =42 °C); these peptides were selected for their similar *T_m_* values. The peptides were heated from 4 °C to 92 °C and then cooled back down to 4 °C at the same rate. With an extreme temperature change rate of 1 °C/min, peptide **3** exhibited a small hysteresis of 1.8±0.2 °C whereas H‐(GPO)_7_‐NH_2_ exhibited a much larger hysteresis over 20 °C (Figure 3H). In addition, peptides **4−6** exhibit similar hysteresis with peptide **3**, ranging from 1.9−2.6 °C. (Figure S4A; Table S2) Therefore, the crosslinking of bis β‐turn CMPs provides an order of magnitude increase in kinetics compared with a typical linear CMP. This intramolecular folding mechanism thus demonstrates more rapid kinetics than the canonical intermolecular mechanism that requires three separate strands to self‐assemble.

**Figure 4:**
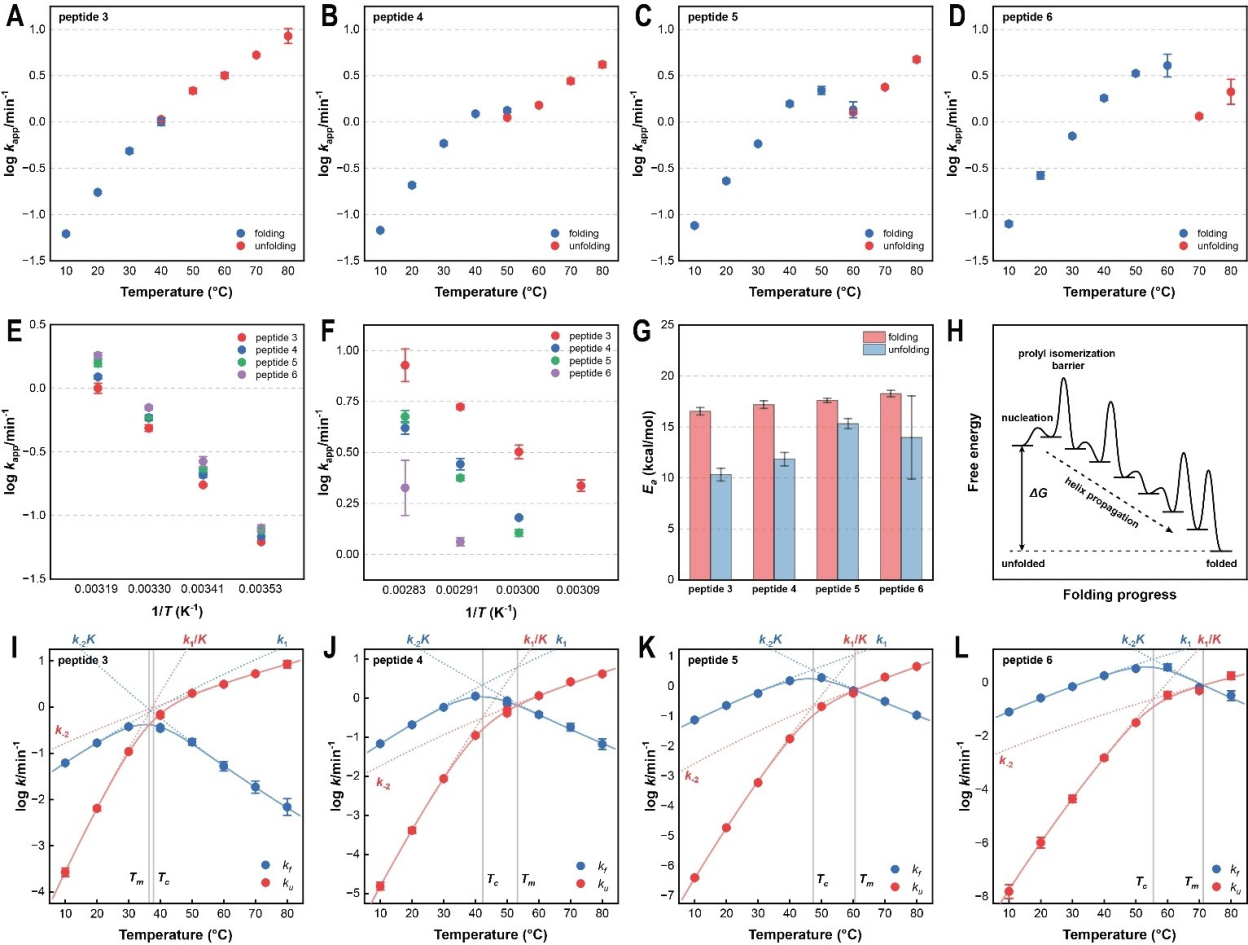
Folding kinetics characterization of bis β‐turn CMPs. (A−D) The apparent folding/unfolding rate constants of peptides **3**–**6**. Blue points were measured by the folding of denatured peptides, whereas red points were obtained from the unfolding of triple helical peptides. (E−F) Plot of the apparent rate constant versus 1/*T* of peptides **3**–**6** at low and high temperatures, respectively. (G) Activation energy for peptide folding and unfolding calculated from the data in graphs (E) and (F). (H) Schematic bis β‐turn CMP free energy diagram of a certain folding pathway. (I−L) Kinetics data fitted to the 2‐step reaction model of peptides **3**–**6**. The rate constants for folding (*k_f_*) and unfolding (*k_u_*) are marked as circles. The fitted curves are displayed as solid lines, and the dotted lines are their extrapolation.

The hysteresis of collagen folding is substantial, and acceleration of collagen folding can be achieved by lipidation or crosslinking.[^64^, ^65^] In previous studies on crosslinked CMPs, fast folding and low hysteresis were observed to a similar degree for β‐turn CMPs.[^41^, ^42^, ^44^, ^45^, ^47^, ^66^] To further investigate the folding kinetics and mechanism of bis β‐turn CMPs, we conducted temperature‐dependent kinetic measurements, which have not been performed over a large temperature range in previous CMP studies. The CD ellipticity signal at 225 nm was monitored at a series of constant temperatures where the peptide folding rate was monitored by cooling a heated, unfolded peptide and vice versa. All CD signal curves exponentially approach the equilibrium state, and the curve can be fitted using the first‐order reversible reaction model to give the apparent rate constant *k_app_* at various temperatures (Figure 4A–D). At temperatures close to *T_m_*, we were able to measure the folding rate by either folding or unfolding the peptide because the peptide is partially folded at equilibrium. Theoretically, the *k_app_* measured by folding and unfolding should be the same since the apparent rate constant equals the sum of folding and unfolding rate constants (*k_app_* =*k_f_* +*k_u_*) in a reversible first‐order reaction.

All peptides show linearity between ln *k_app_* and 1/*T* at low and high temperatures, consistent with the Arrhenius equation (Figure 4E, F). At low temperatures, longer peptides give slightly faster kinetics, and at high temperatures, longer peptides behave more slowly. At 30 °C, the measured *k_app_* for peptides **3**–**6** are 0.45 min^−1^, 0.58 min^−1^, 0.58 min^−1^, and 0.7 min^−1^, respectively, corresponding to a half‐time of 1.5 to 1 min. Linear regression between ln *k_app_* and 1/*T* in the low‐temperature and high‐temperature regions yields the apparent activation energy for bis β‐turn CMP folding and unfolding (Figure 4G). All peptides **3**–**6** fold with a similar activation energy from the 10–40 °C data that slightly increases from 16.8 to 18.0 kcal/mol as the peptide length grows. Unfolding activation energy values range from 10.4 to 15.3 kcal/mol and exhibit more significant increases as the peptide lengthens. However, an exception is observed for peptide **6**, which may be caused by the large error in the kinetics measurement at high temperatures.

Although the CD folding and unfolding signals precisely fit into a reversible first‐order reaction model and the Chevron plot of isothermal kinetics data (Figure S5) is consistent with a two‐state protein folding model, the complexity of bis β‐turn CMP folding becomes apparent when we investigate their temperature‐dependent folding behavior. The log *k_app_* curve exhibits one or multiple inflection points as temperature increases, and at temperatures close to *T_m_*, there are significant dips in the log *k_app_* value, especially for longer peptides. This result implies that bis β‐turn CMP folding has a multi‐step mechanism (Figure 4H), and the rate‐limiting step is different at low and high temperatures.

Combining the kinetics data with the CD thermodynamics data allows us to deconvolute the folding and unfolding rate constants (*k_f_* and *k_u_*) from the apparent rate constant *k_app_* at all temperatures. The folding equilibrium constant *K* can be calculated from the fitted *ΔH* and *ΔS* values, and *k_f_* and *k_u_* can be derived from the following equation: *K*=*k_f_*/*k_u_* and *k_app_* =*k_f_* +*k_u_*. Therefore, we can obtain the unfolding kinetics at low temperatures and folding kinetics at high temperatures (Figure 4I–L). For all peptides, the plot of calculated *k_f_* and *k_u_* as a function of *T* shows two limbs and an obvious inflection point between them, indicating that there is a possible rate‐limiting step change at a critical temperature, *T_c_*.

Prior studies have proposed mechanisms for collagen folding, which generally involve nucleation of the triple helix and zipper‐like helix propagation.[^67^, ^68^, ^69^, ^70^] Proline *cis‐trans* isomerization is thought to play a critical role in determining the collagen folding rate.^[71]^ The helix propagation combined with the prolyl isomerization results in a multistep folding mechanism with multiple pathways. The *trans*‐proline has preorganized conformations resulting in a lower barrier for triple helix formation, while the *cis*‐proline must isomerize to a *trans* configuration by overcoming a high energy barrier (Figure 4H). In the bis β‐turn CMP, the nucleation of the triple helix is significantly promoted by the proximity provided by crosslinking. Therefore, the prolyl isomerization is likely to be rate‐limiting.

(1) $$U\;\underset{k_{-1}}{\overset{k_{1}}{\vec{\leftarrow }}}\;{I\;}_{1}\underset{k_{-2}}{\overset{k_{2}}{\vec{\leftarrow }}}\;{I\;}_{2}\;\cdots \;{I\;}_{n-1}\;\underset{k_{-n}}{\overset{k_{n}}{\vec{\leftarrow }}}\;F$$

(2) $$U\;\underset{k_{-1}}{\overset{k_{1}}{\vec{\leftarrow }}}\;I\;\underset{k_{-2}}{\overset{k_{2}}{\vec{\leftarrow }}}\;F$$

(3) $$k_{app}=\;\frac{k_{1}k_{-2}\left(K+1\right)}{k_{1}+k_{-2}K}\;$$

To investigate the temperature‐dependent folding kinetics of bis β‐turn CMPs, we proposed a multistep mechanism of collagen folding in which the elementary steps include prolyl isomerization, helix nucleation, and helix propagation (Equation 1). As we cannot deconvolute the order and the kinetics of all steps, we found that simplifying the mechanism into a two‐step reversible reaction model results in adequate fitting of the experimental data. The two‐step reaction involves a simplified intermediate **I** between unfolded peptide **U** and folded peptide **F** (Equation 2). Applying the steady‐state assumption, we can derive the formula for *k_app_* (Equation 3), folding rate constant *k_f_* and unfolding rate constant *k_u_* (Supporting Information), which are functions of the rate constant of the first forward step (*k*_1_), the second backward step (*k*_‐2_), and the folding‐unfolding equilibrium constant *K*. As we can calculate *K* from the fitting of the CD data, building a model of the temperature dependency of *k*_1_ and *k*_‐2_ allows us to solve the kinetics. Using an Arrhenius model, defining ln *k*_1_=*A*+*E*_a,1_/*RT* and ln *k*_‐2_=*B*+*E*_a,‐2_/*RT*, yields good non‐linear fitting to the experimental data (*k_f_* and *k_u_* fitting in Figure 4I–L; *k_app_* fitting in Figure S6).

Calculating ‐*R*(*d*ln*k/dT*) would afford the apparent activation energy of forward step 1 (*E*_a,1_) and backward step 2 (*E*_a,‐2_) but due to the unknown stability of intermediate **I**, the *E*_a,2_ and *E*_a,‐1_ values were not deconvoluted (Table 2). The calculated activation energy for the first folding steps of peptides **3**–**6** ranges between 16.2 and 19.2 kcal/mol, which closely approximates the activation energy of proline *cis‐trans* isomerization (~20 kcal/mol).^[72]^ This similarity leads to the hypothesis that proline isomerization may be the rate‐limiting step in peptide folding at lower temperatures, as previously reported.[^71^, ^72^, ^73^] Therefore, in combination with the fitting results, we can propose a folding mechanism for bis β‐turn CMPs. At low temperatures with *T* < *T_c_*, the slow kinetics of proline *cis‐trans* isomerization makes it the rate‐limiting step of bis β‐turn CMP folding. The proximity provided by the linkers makes nucleation much faster in bis β‐turn CMPs, but zipper‐like helix propagation is hindered when *cis*‐proline is present. Consequently, the peptide triple helix is rapidly nucleated but grows slowly with the growth rate coupled to the proline *cis‐trans* isomerization rate. With an increase in temperature when *T* > *T_c_*, prolyl *cis‐trans* isomerization is faster, the stability of partially folded nuclei is reduced, and helix propagation is less favorable. As a result, the concentration of the partially folded intermediate is reduced, leading to a decline in the overall folding rate. Therefore, the rate‐limiting step switches to triple helix nucleation and helix growth at high temperatures.

**Table 2:** Results of kinetics data fitted into the two‐step reversible reaction model.

Peptide	*T_c_* (°C)	*T_m_* – *T_c_* (°C)	*k*_1_, _10 °C_ (min^−1^)	*E*_a,1_ (kcal/mol)	*E*_a,‐2_ (kcal/mol)
**3**	37.8	−1.3	0.063	16.2±0.6	10.7±0.4
**4**	42.3	11.0	0.065	19.2±1.1	15.4±1.0
**5**	47.3	13.3	0.071	19.2±1.5	20.5±2.1
**6**	55.5	15.8	0.085	18.1±1.7	17.2±5.1

We can calculate the critical temperature *T_c_* where the first and second steps contribute equally to the overall kinetics,^[74]^ and we can derive that *k*_*‐*1_ = *k*_2_ and *k*_1_ = *k*_‐2_ *K* at *T_c_*. Peptides with differences in length have distinct *T_c_* values, and the *T_m_*–*T_c_* value can be used to characterize the contribution of nucleation and helix propagation in peptide folding. All peptides **3**−**6** exhibit similar *k*_1_ at low temperatures (Table 2), indicating that the proline isomerization contributes similarly to peptide folding. Additionally, longer peptides have larger *T_m_* − *T_c_* values, which suggests nucleation and helix propagation are more favorable and contribute more to the kinetics as the peptide length increases.

## Conclusions

In summary, we have designed and prepared a series of linearly turn‐linked, monomeric collagen mimetic peptides, termed bis β‐turn CMPs, connected by minimal β‐turn mimicking linkers. With advances in well‐defined structural design and improved synthetic efficiency, this new turn‐linking strategy effectively increases triple helix thermal stability and allows for miniaturization of the triple helix. The shortest stable triple helix is 7 residues in length in TMAO conditions and 10 residues in PBS buffer. The folding mechanism of bis β‐turn CMPs is intramolecular, which substantially accelerates the folding kinetics. Bis β‐turn CMPs are heterotrimeric consisting of three frame‐shifted strands, which are expected to be compatible with the incorporation of functional sequences to construct protein‐targeting collagen heterotrimers. Our comprehensive characterization of bis β‐turn CMP folding kinetics provides novel insights regarding folding, where both triple helix formation and proline *cis‐trans* isomerization contribute to the apparent folding rates in a simplified two‐step model. Bis β‐turn CMPs, with their strong stability, small size, and rapid folding kinetics, hold promise for a new generation of collagen model peptides that can be used as ligands or building blocks in chemical biology and bioengineering studies.

## Supporting Information

The authors have cited additional references within the Supporting Information.[^61^, ^75^, ^76^, ^77^, ^78^]

## Conflict of Interests

The authors declare no conflict of interest.

## Supporting information

As a service to our authors and readers, this journal provides supporting information supplied by the authors. Such materials are peer reviewed and may be re‐organized for online delivery, but are not copy‐edited or typeset. Technical support issues arising from supporting information (other than missing files) should be addressed to the authors.

Supporting Information

## Data Availability

The data that support the findings of this study are available in the supplementary material of this article.
