# Supplementary material for: β‐Turn Mimicking Crosslinking Provides Hyperstability and Fast Folding Kinetics for Short Collagen Triple Helices
Source: Chembiochem. 2025 Mar 19;26(12):e202400834. doi: 10.1002/cbic.202400834 (PMC12177705; doi:10.1002/cbic.202400834)
Supplement: Supplementary file 1 — Supporting Information [file CBIC-26-e202400834-s001.pdf]

ChemBioChem

Supporting Information

β-Turn Mimicking Crosslinking Provides Hyperstability and Fast Folding Kinetics for Short Collagen Triple Helices

Pengfei Jin, Diane N. Rafizadeh, Huanyi Zhao, and David M. Chenoweth*

β-Turn Mimicking Crosslinking Provides Hyperstability and Fast Folding Kinetics for Short Collagen Triple Helices

Pengfei Jin, Diane N. Rafizadeh, Huanyi Zhao and David M. Chenoweth*

Department of Chemistry, University of Pennsylvania, Philadelphia, Pennsylvania 19104, United States

*Email: dcheno@sas.upenn.edu

SUPPORTING INFORMATION

Table of Contents

Supplementary Figures....S02

Supplementary Tables....S05

Reagents....S12

Instrumentation....S13

Peptide Synthesis and Purification....S14

Circular Dichroism Spectroscopy....S17

Differential Scanning Calorimetry....S18

Size Exclusion Chromatography....S18

Thermodynamic Model of Bis β-Turn CMP folding....S19

Kinetics Model of Bis β-Turn CMP folding....S21

Supplementary Data....S22

References....S50

SUPPLEMENTARY FIGURES

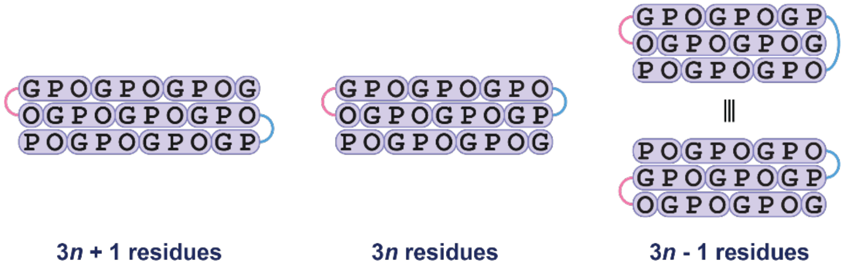

Figure S1. Determining the sequences of bis β-turn CMPs. The β-turn mimicking linkers have fixed configurations, in which Gly and Hyp are connected at the N-terminus whereas Hyp and Pro are connected at the C-terminus. Dictated by the fixed connections, a peptide 3*n* residues in helix length is not accessible as a single crosslinked molecule.

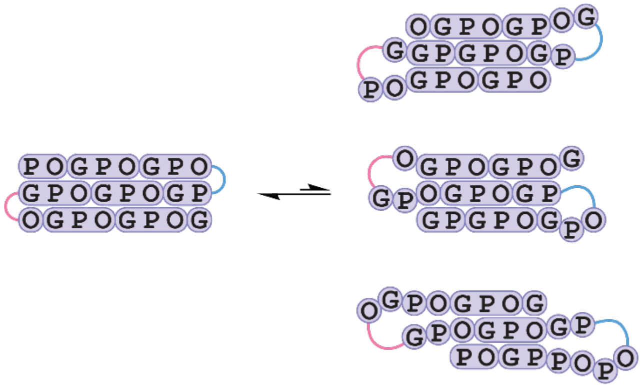

Figure S2. Possible folding side products of a bis β-turn CMP. The misaligned triple helices have shorter, less stable triple helical regions and flexible, unoptimized loops. These alternative helical states are considered unstable and have minimal contribution to bis β-turn CMP folding.

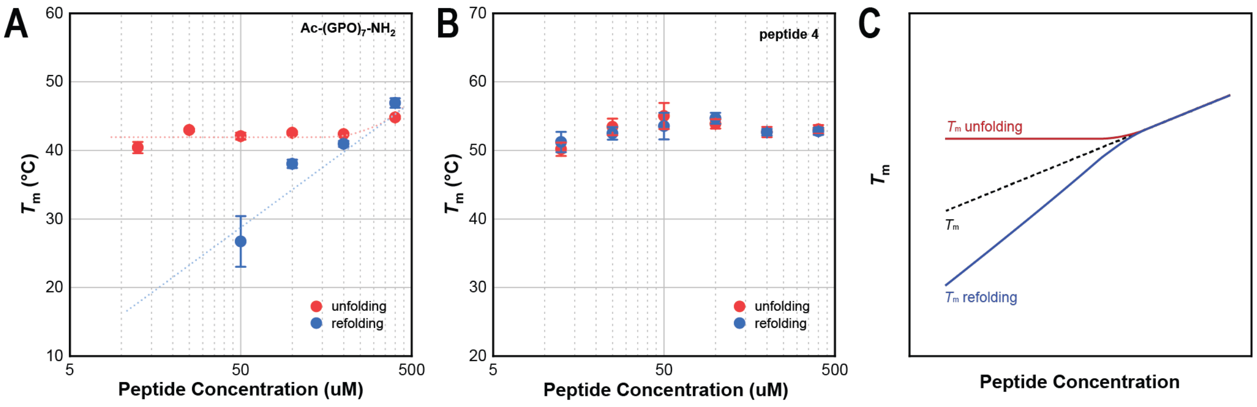

Figure S3. Concentration dependence in the folding of Ac-(GPO)$_7$-NH$_2$ and peptide 4. (A and B) Concentration dependent unfolding and refolding data of Ac-(GPO)$_7$-NH$_2$ and peptide **4**. Data collected with a slow heating and colling rate of 0.05 ºC/min. (C) Hypothetical data of concentration dependent melting temperature of trimeric CMP folding and unfolding. T_m

measured at low concentration (red and blue curve) exhibit deviation to the equilibrium T_m value (black line) due to high hysteresis.

Ac-$(GPO)_7$-NH_2 exhibited limited temperature dependency in the melting experiment, while the refolding experiment indicates a significant decrease in T_m when the peptide concentration is reduced. (Figure S3A) This complexity is due to the extremely high hysteresis at low peptide concentration, where the peptide was not able to reach equilibrium during the unfolding and refolding experiments. The T_m, if the peptide is allowed to reach the equilibrium, should be a value between $T_{m,\ unfolding}$ and $T_{m,\ refolding}$ with a concentration dependency. (Figure S3C) In contrast, peptide **4** exhibited no hysteresis and similar T_m at all concentrations, due to its intramolecular folding mechanism.

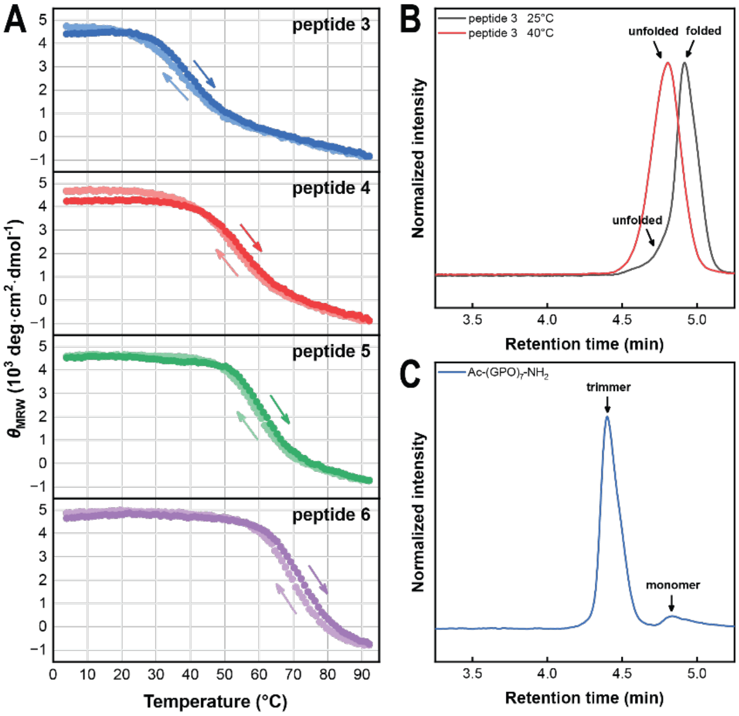

Figure S4. Supplementary characterization data. (A) Hysteresis measurement of peptides **3**–**6**. (B) SEC trace of peptide **3** at room temperature (approximately 25 °C) and 40 °C. (C) SEC trace of Ac-$(GPO)_7$-NH_2 at room temperature.

Supporting data by SEC confirms that bis β-turn CMP folding is intramolecular. At room temperature, peptide 3 and Ac-$(GPO)_7$-NH_2 exhibit only small populations of the unfolded state, as they have T_m values of 36 °C and 48 °C, respectively. For peptide 3, the unfolded state can be better visualized by SEC upon heating to 40 °C. In Figure S3B, the unfolded peptide **3** has a slightly shorter retention time and a broader peak than the folded peptide, as the hydrodynamic diameter of the random coil is larger than the compact triple helix. In contrast, the unfolded (monomeric) Ac-$(GPO)_7$-NH_2 has a longer retention time than the folded (trimeric) peptide (Figure S3C) due to the difference in molecular weight between the monomer and trimer.

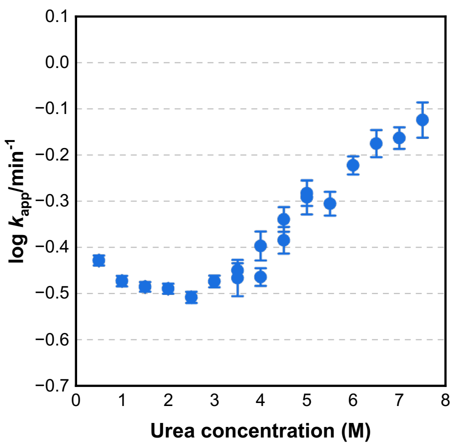

Figure S5. Isothermal kinetics measurement of bis β-turn CMP folding. The orange lines are fitted curves using the two-step folding model.

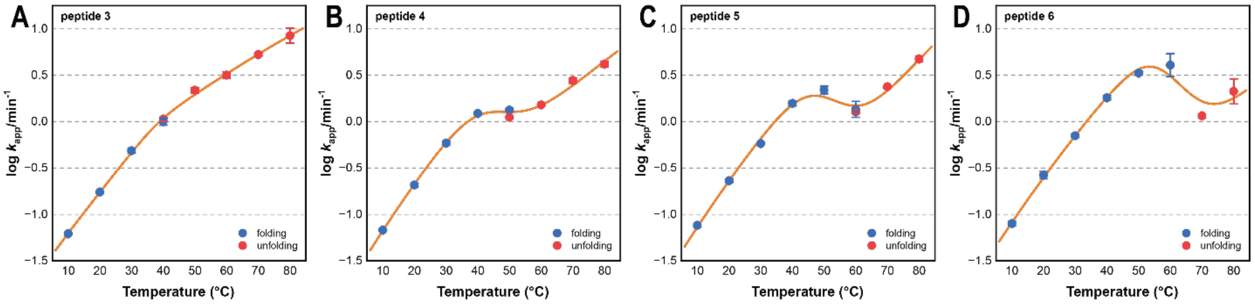

Figure S6. Fitting results of the bis β-turn CMP kinetics. The orange lines are fitted curves using the two-step folding model.

SUPPLEMENTARY TABLES

Table S1. CD thermal denaturation fitting results

Peptide	T_m (°C)	ΔH (kJ mol^{-1})	ΔS (J mol^{-1} K^{-1})
Peptide 3	36.77 ± 0.11	-142.5 ± 2.0	-460 ± 6
Peptide 3	36.28 ± 0.14	-149.8 ± 2.9	-484 ± 9
Peptide 3	36.35 ± 0.16	-155.9 ± 3.6	-504 ± 12
Peptide 4	52.46 ± 0.13	-152.2 ± 2.5	-467 ± 8
Peptide 4	54.02 ± 0.16	-141.7 ± 2.4	-433 ± 7
Peptide 4	53.21 ± 0.13	-152.3 ± 2.4	-467 ± 7
Peptide 5	60.71 ± 0.11	-187.3 ± 2.4	-561 ± 7
Peptide 5	60.81 ± 0.08	-187.1 ± 1.9	-560 ± 6
Peptide 5	60.21 ± 0.09	-191.5 ± 2.3	-575 ± 7
Peptide 6	72.25 ± 0.28	-190.5 ± 3.3	-552 ± 10
Peptide 6	69.85 ± 0.22	-214.7 ± 4.3	-626 ± 12
Peptide 6	71.80 ± 0.31	-202.3 ± 4.5	-587 ± 13
peptide 1 in 1M TMAO	7.96 ± 1.35	-143.1 ± 19.4	-509 ± 69
peptide 1 in 2M TMAO	12.29 ± 0.68	-127.2 ± 10.5	-446 ± 37
peptide 1 in 2.5M TMAO	15.14 ± 0.42	-136.1 ± 8.8	-472 ± 31
peptide 1 in 3M TMAO	16.86 ± 0.56	-122.5 ± 9.9	-422 ± 34
peptide 1 in 3.5M TMAO	20.49 ± 0.46	-119.4 ± 8.4	-407 ± 29
peptide 1 in 4M TMAO	21.23 ± 0.66	-104.5 ± 9.5	-355 ± 32
peptide 2 in 1M TMAO	25.24 ± 0.87	-134.9 ± 9.8	-452 ± 33
peptide 2 in 1.5M TMAO	27.47 ± 0.83	-100.3 ± 5.4	-334 ± 18
peptide 2 in 2M TMAO	32.83 ± 0.67	-101.2 ± 5.6	-331 ± 18
peptide 2 in 2.5M TMAO	36.58 ± 0.60	-108.3 ± 6.6	-350 ± 21
peptide 2 in 3M TMAO	40.84 ± 0.63	-106.1 ± 7.1	-338 ± 23
peptide 2 in 3.5M TMAO	45.78 ± 0.59	-136.8 ± 10.4	-429 ± 33
peptide 2 in 4M TMAO	45.59 ± 1.00	-97.57 ± 9.1	-306 ± 29
H-(GPO)$_7$-NH$_2$	43.83 ± 0.07	-529.6 ± 9.8	-1654 ± 31
H-(GPO)$_7$-NH$_2$	42.01 ± 0.08	-492.2 ± 9.2	-1544 ± 29
H-(GPO)$_7$-NH$_2$	42.18 ± 0.09	-492.4 ± 10.8	-1544 ± 34
Ac-(GPO)$_7$-NH$_2$	47.41 ± 0.06	-623.2 ± 10.9	-1927 ± 34
Ac-(GPO)$_7$-NH$_2$	47.06 ± 0.06	-588.0 ± 11.0	-1819 ± 34
Ac-(GPO)$_7$-NH$_2$	48.85 ± 0.05	-665.2 ± 10.5	-2048 ± 33
Ac-(GPO)$_9$-NH$_2$	64.69 ± 0.04	-821.8 ± 11.2	-2415 ± 33
Ac-(GPO)$_9$-NH$_2$	65.18 ± 0.04	-827.5 ± 11.3	-2429 ± 33
Ac-(GPO)$_9$-NH$_2$	64.72 ± 0.03	-882.7 ± 10.3	-2595 ± 30
Peptide 3, c = 12.5 μmol	35.75 ± 0.37	-140.2 ± 6.5	-454 ± 21
Peptide 3, c = 25 μmol	36.00 ± 0.18	-142.1 ± 3.4	-460 ± 11
Peptide 3, c = 50 μmol	36.08 ± 0.34	-136.9 ± 5.9	-443 ± 19
Peptide 3, c = 100 μmol	36.72 ± 0.13	-142.7 ± 2.5	-460 ± 8

Peptide 3, c = 400 µmol	36.74 ± 0.08	-141.1 ± 1.5	-455 ± 5
Peptide 4, c = 12.5 µmol	52.50 ± 0.45	-143.2 ± 6.9	-440 ± 21
Peptide 4, c = 25 µmol	53.45 ± 0.24	-152.7 ± 4.2	-468 ± 13
Peptide 4, c = 50 µmol	54.46 ± 0.31	-137.5 ± 4.2	-420 ± 13
Peptide 4, c = 100 µmol	52.72 ± 0.24	-140.6 ± 3.6	-432 ± 11
Peptide 4, c = 400 µmol	52.94 ± 0.13	-148.2 ± 2.3	-455 ± 7
Peptide 5, c = 12.5 µmol	60.45 ± 0.20	-188.0 ± 4.5	-564 ± 14
Peptide 5, c = 25 µmol	60.24 ± 0.14	-191.6 ± 3.3	-575 ± 10
Peptide 5, c = 50 µmol	60.89 ± 0.20	-181.3 ± 4.0	-543 ± 12
Peptide 5, c = 100 µmol	59.62 ± 0.11	-201.2 ± 3.1	-604 ± 9
Peptide5, c = 400 µmol	59.26 ± 0.10	-196.7 ± 2.8	-592 ± 8
Peptide 6, c = 12.5 µmol	72.72 ± 0.32	-195.6 ± 4.0	-566 ± 11
Peptide 6, c = 25 µmol	72.36 ± 0.28	-194.3 ± 3.5	-562 ± 10
Peptide 6, c = 50 µmol	72.01 ± 0.34	-209.0 ± 5.4	-606 ± 16
Peptide 6, c = 100 µmol	70.12 ± 0.20	-216.2 ± 4.1	-630 ± 12
Peptide 6, c = 400 µmol	70.26 ± 0.22	-217.0 ± 4.3	-632 ± 13

Table S2. CD hysteresis results

CD measurement	T_m (°C)	Hysteresis (°C)
Peptide 3 unfolding	36.59 ± 0.45	1.8 ± 0.5
Peptide 3 refolding	38.34 ± 0.30	
Peptide 4 unfolding	51.71 ± 0.38	2.4 ± 0.5
Peptide 4 refolding	54.09 ± 0.39	
Peptide 5 unfolding	58.73 ± 0.30	1.9 ± 0.5
Peptide 5 refolding	60.62 ± 0.36	
Peptide 6 unfolding	70.16 ± 0.54	2.6 ± 0.9
Peptide 6 refolding	72.73 ± 0.66	

Table S3. DSC fitting results

Peptide	E_f (kcal mol^{-1} K^{-1})	D_f (kcal mol^{-1} K^{-2})	E_u (kcal mol^{-1} K^{-1})	D_u (kcal mol^{-1} K^{-2})	ΔH (kcal mol^{-1})	T_m (°C)	c_{fit}/c_{sample}
3	-1.0031 ± 0.1643	0.003683 ± 0.000602	-3.3197 ± 0.1876	0.006254 ± 0.000537	-30.36 ± 0.25	33.48 ± 0.10	0.907 ± 0.014
4	5.4097 ± 0.0965	-0.02206 ± 0.000336	7.1386 ± 0.1792	-0.02557 ± 0.000477	-35.18 ± 0.20	55.73 ± 0.18	0.976 ± 0.009
5	-5.0292 ± 0.05056	0.013476 ± 0.000173	-6.4298 ± 0.1142	0.015031 ± 0.000308	-44.95 ± 0.20	59.20 ± 0.08	0.991 ± 0.010
6	-3.2536 ± 0.0321	0.007864 ± 0.000108	-3.4793 ± 0.2915	0.005969 ± 0.000600	-51.21 ± 0.71	71.56 ± 0.11	0.922 ± 0.043

Table S4. SEC results (RT: room temperature, approx. 25 ºC). Retention times represent results from runs in triplicate.

Peptide	Folding type	Temperature (°C)	Retention time (min)			Helix length	M (g mol^{-1})	log M
3	monomeric	RT	4.918	4.917	4.917	10	2988.2	3.475
4	monomeric	RT	4.860	4.845	4.848	11	3255.5	3.513
5	monomeric	RT	4.762	4.763	4.762	13	3790.0	3.579
6	monomeric	RT	4.702	4.688	4.690	14	4057.3	3.608
3	monomeric	40	4.808	4.805	4.798	10	2988.2	3.475
Ac-(GPO)$_7$-NH$_2$	trimeric	RT	4.400	4.400	4.402	21	5790.2	3.763
Ac-(GPO)$_9$-NH$_2$	trimeric	RT	4.142	4.143	4.143	27	7393.9	3.869

Table S5. Isothermal kinetics characterization of peptide **4**

Experiment	Urea concentration (M)	k (min^{-1})
folding	0.5	-0.4284 ± 0.0108
folding	1	-0.4729 ± 0.0113
folding	1.5	-0.4853 ± 0.0105
folding	2	-0.4894 ± 0.0109
folding	2.5	-0.5081 ± 0.0121
folding	3	-0.4737 ± 0.0126
folding	3.5	-0.4498 ± 0.0155
folding	4	-0.4642 ± 0.0190
folding	4.5	-0.3397 ± 0.0266
folding	5	-0.2921 ± 0.0368
unfolding	3.5	-0.4664 ± 0.0391
unfolding	4	-0.3971 ± 0.0312
unfolding	4.5	-0.3847 ± 0.0280
unfolding	5	-0.2833 ± 0.0273
unfolding	5.5	-0.3058 ± 0.0256
unfolding	6	-0.2075 ± 0.0282
unfolding	6	-0.2464 ± 0.0274
unfolding	6.5	-0.1760 ± 0.0294
unfolding	7	-0.2033 ± 0.0345
unfolding	7	-0.1249 ± 0.0316
unfolding	7.5	-0.1250 ± 0.0381

Table S6. Kinetics characterization results of peptides **3–6**. Data with the temperature values colored in blue and red were used in calculating $E_{a,\ folding}$ and $E_{a,\ folding}$, respectively.

Peptide	Experiment	T (°C)	k (min^{-1})		
3	folding	10	0.06234 ± 0.00042	0.06297 ± 0.00074	0.06037 ± 0.00070
3	folding	20	0.1778 ± 0.0018	0.1732 ± 0.0025	0.1697 ± 0.0024
3	folding	30	0.4529 ± 0.0111	0.4981 ± 0.0154	0.5051 ± 0.0155
3	folding	40	1.105 ± 0.052	0.9406 ± 0.0398	0.9564 ± 0.0367
3	unfolding	40	1.119 ± 0.024	1.054 ± 0.026	1.023 ± 0.027
3	unfolding	50	2.332 ± 0.047	2.119 ± 0.034	2.065 ± 0.034
3	unfolding	60	3.323 ± 0.111	2.897 ± 0.091	3.319 ± 0.104
3	unfolding	70	5.141 ± 0.236	5.286 ± 0.306	5.435 ± 0.277
3	unfolding	80	9.728 ± 0.962	8.943 ± 0.933	6.724 ± 0.486
4	folding	10	0.06740 ± 0.00056	0.06802 ± 0.00152	0.06748 ± 0.00167
4	folding	20	0.1975 ± 0.0019	0.2075 ± 0.0032	0.2166 ± 0.0038
4	folding	30	0.5543 ± 0.0137	0.6117 ± 0.0210	0.5915 ± 0.0229
4	folding	40	1.235 ± 0.045	1.261 ± 0.050	1.181 ± 0.051
4	folding	50	1.403 ± 0.099	1.308 ± 0.059	1.287 ± 0.062
4	unfolding	50	1.113 ± 0.033	1.108 ± 0.034	1.126 ± 0.040
4	unfolding	60	1.531 ± 0.019	1.489 ± 0.022	1.520 ± 0.028
4	unfolding	70	2.601 ± 0.049	2.960 ± 0.067	2.752 ± 0.054
4	unfolding	80	3.852 ± 0.127	4.438 ± 0.198	4.212 ± 0.158
5	folding	10	0.07578 ± 0.00050	0.07908 ± 0.00071	0.07363 ± 0.00066
5	folding	20	0.2245 ± 0.0029	0.2245 ± 0.0037	0.2426 ± 0.0043
5	folding	30	0.5836 ± 0.0125	0.5819 ± 0.0160	0.576 ± 0.0169
5	folding	40	1.481 ± 0.035	1.670 ± 0.044	1.555 ± 0.054
5	folding	50	1.982 ± 0.050	2.191 ± 0.054	2.402 ± 0.057
5	folding	60	1.666 ± 0.149	1.237 ± 0.083	1.170 ± 0.071
5	unfolding	60	1.324 ± 0.021	1.277 ± 0.021	1.223 ± 0.021
5	unfolding	70	2.292 ± 0.027	2.402 ± 0.036	2.423 ± 0.033
5	unfolding	80	4.730 ± 0.100	5.061 ± 0.098	4.415 ± 0.098
6	folding	10	0.08442 ± 0.00052	0.07573 ± 0.00088	0.07803 ± 0.00078
6	folding	20	0.2631 ± 0.0060	0.2894 ± 0.0087	0.2423 ± 0.0084
6	folding	30	0.7257 ± 0.0264	0.6727 ± 0.0371	0.7129 ± 0.0337
6	folding	40	1.799 ± 0.062	1.720 ± 0.094	1.911 ± 0.096
6	folding	50	3.530 ± 0.123	3.198 ± 0.120	3.290 ± 0.128
6	folding	60	3.044 ± 0.129	3.842 ± 0.208	5.321 ± 0.257
6	unfolding	70	1.182 ± 0.024	1.093 ± 0.022	1.184 ± 0.023
6	unfolding	80	1.761 ± 0.031	1.720 ± 0.029	2.879 ± 0.079

Table S7. Linear regression results for calculating the folding/unfolding activation energy of peptides **3**–**6**.

Peptide	$E_{a,\ folding}$ (kcal mol^{-1})	$E_{a,\ unfolding}$ (kcal mol^{-1})
3	16.53 ± 0.36	10.32 ± 0.63
4	17.18 ± 0.37	11.84 ± 0.65
5	17.61 ± 0.22	15.32 ± 0.51
6	18.27 ± 0.31	13.97 ± 4.09

Table S8. Nonlinear fitting results of the peptide folding kinetics using the 2-step model.

Peptide	T_c (°C)	$E_{a,\ folding}$ (kcal mol^{-1})	$E_{a,\ unfolding}$ (kcal mol^{-1})
3	37.22	16.53 ± 0.86	11.17 ± 0.58
4	42.07	19.19 ± 1.04	15.54 ± 0.97
5	47.15	18.92 ± 1.46	21.13 ± 2.13
6	55.34	18.39 ± 1.72	17.34 ± 5.05

ABBREVIATIONS

Boc	tert-Butyloxycarbonyl
CD	Circular dichroism
CHCA	α-Cyano-4-hydroxycinnamic acid
CMP	Collagen mimetic peptide
DBU	1,8-Diazabicyclo[5.4.0]undec-7-ene
DCM	Dichloromethane
DMF	*N*,*N*-Dimethylformamide
DIEA	*N*,*N*-Diisopropylethylamine
DSC	Differential scanning calorimetry
Fmoc	9-Fluorenylmethoxycarbonyl
HBTU	3-[Bis(dimethylamino)methyliumyl]-3H-benzotriazol-1-oxide hexafluorophosphate
HOBt	1-Hydroxybenzotriazole
HPLC	High-performance liquid chromatography
MALDI-TOF MS	Matrix-assisted laser desorption/ionization mass spectrometry
NMP	N-Methyl-2-pyrrolidone
PBS	Phosphate-buffered saline
PyAOP	(7-Azabenzotriazol-1-yloxy)tripyrrolidinophosphonium hexafluorophosphate
PyOxim	[Ethyl cyano(hydroxyimino)acetato-O^2]tri-1-pyrrolidinylphosphonium hexafluorophosphate
RT	Room temperature
SEC	Size-exclusion chromatography
SPPS	Solid-phase peptide synthesis
tBu	tert-Butyl
TFA	Trifluoroacetic acid
TIPS	Triisopropylsilane
UPLC-MS	Ultra-high performance liquid chromatography-mass spectrometry

REAGENTS

All commercially available reagents were used as received.

Solvents:	Supplier:
Acetonitrile	Fisher
DCM	Fisher
DMF	Fisher
Diethyl ether	Fisher
DCM	Fisher
Methanol	Fisher
NMP	Acros Organics
Toluene	Acros Organics
10X PBS	Fisher

Solvents

DCM, DMF, acetonitrile, methanol, toluene, NMP

Peptide synthesis reagents:	Supplier:
Rink amide MBHA resin	Novabiochem
2-chlorotrityl chloride resin	Aapptec
PyAOP	Chem-Impex Int'l. Inc.
PyOxim	Chem-Impex Int'l. Inc.
DIEA	Chem-Impex Int'l. Inc.
DBU	TCI America
HOBt	Acros Organics
N-Fmoc-ethylenediamine hydrochloride	Chem-Impex Int'l. Inc.
Succinic anhydride	Sigma-Aldrich
TFA	Acros Organics
TIPS	TCI America

Fmoc-Amino Acids:	Supplier:
Fmoc-Pro-OH	Chem-Impex Int'l. Inc.
Fmoc-Hyp(tBu)-OH	AstaTech Inc.
Fmoc-Gly-OH	Chem-Impex Int'l Inc.

Other reagents:	Supplier:
CHCA	Sigma-Aldrich

INSTRUMENTATION

All peptides were purified using preparative or semi-preparative reversed-phase HPLC on an Agilent 1260 Infinity II system using a Phenomenex Aeris 5 µm PEPTIDE XB-C18 100 Å LC Column. Varying gradients of acetonitrile and 0.1% TFA in H_2O were used depending on the individual peptide. Analytical HPLC (spectra shown for each peptide) to check purity was carried out on an Agilent 1260 Infinity II system using a Phenomenex Aeris 5 µm PEPTIDE XB-C18 100 Å LC Column. Mass spectrometry was performed using a Bruker Microflex MALDI-TOF MS mass spectrometer and CHCA as the matrix. Peptides were lyophilized using a Labconco FreeZone Plus 12 Liter Cascade Console Freeze Dry system. UV-Vis absorption spectrophotometry was performed using a Jasco V-650 Spectrophotometer equipped with a PAC-743R multichannel Peltier and 1 cm path-length quartz cells. Circular dichroism experiments were performed using a Jasco J-1500 Circular Dichroism Spectrometer and 1 mm quartz cuvettes. Differential scanning calorimetry was performed using Waters Nano DSC. Size exclusion chromatography was performed using Waters Acquity Ultra Performance LC liquid chromatography-mass spectrometry system equipped with Waters Acquity UPLC Protein BEH SEC Column (125 Å, 1.7 µm, 4.6 mm x 150 mm).

PEPTIDE SYNTHESIS AND PURIFICATION

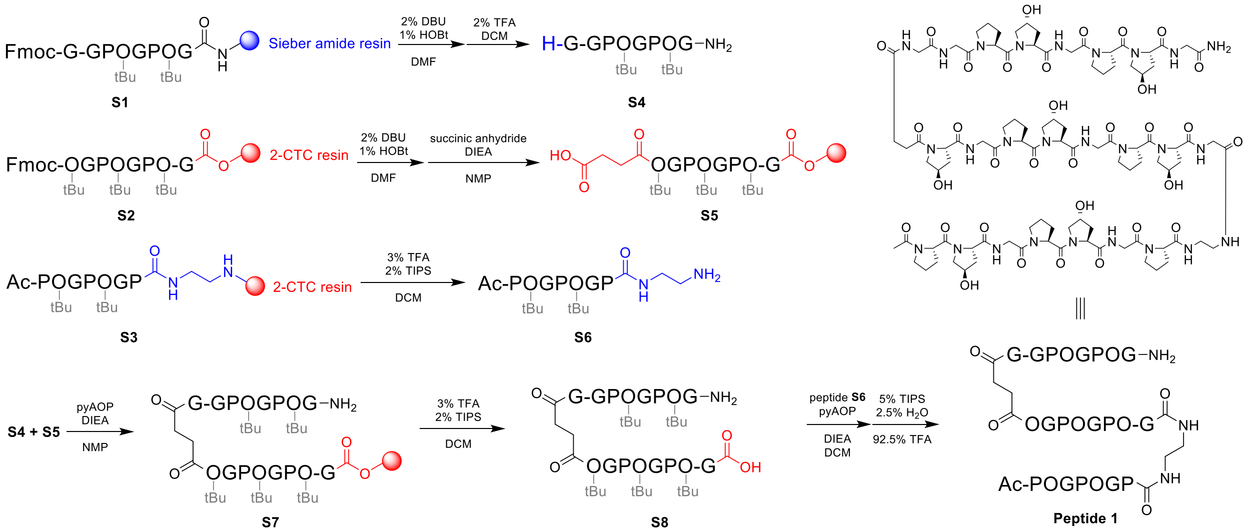

The synthesis of peptides **1**, **3** and **5** involved different fragment coupling reactions than peptides **2**, **4** and **6** (shown in the article in Figure 2).

Preparation of dipeptide and tripeptide building blocks

Fmoc-Gly-Pro-Hyp(tBu)-OH, Fmoc-Pro-Hyp(tBu)-OH, and Fmoc-Gly-Pro-OH were synthesized using methods previously described by our group.[1, 2]

Resin Preparation

All peptides in this study were prepared on a 0.03-0.05 mmol scale on Sieber Amide Resin (loading density: 0.61 mmol/g) and 2-chlorotrityl chloride resin (loading density: 1.48 mmol/g).

Load Fmoc-Gly-OH to 2-chlorotrityl chloride resin

75 mg of resin (3.7 eq to the peptide) was transferred into a 10 mL SPPS vessel. The resin was allowed to swell in anhydrous DCM for a period of 1-2 min before being drained. 1 equivalent (0.03 mmol) of Fmoc-Gly-OH and 7.4 equivalents of DIEA were dissolved in anhydrous DCM and added to the resin. The combination was stirred at RT for 30 min, followed by the addition of 100 μL of MeOH and an additional 15 min of stirring. The solution was then drained, the resin was washed with DMF, and the Fmoc group was removed using the standard protocol (described below).

Load ethylenediamine to 2-chlorotrityl chloride resin

75 mg of resin (3.7 eq relative to the peptide) was transferred into a 10 mL SPPS vessel. The resin was allowed to swell in anhydrous DCM for a period of 1-2 min before being drained. 1 eq (0.03 mmol) of N-Fmoc-ethylenediamine hydrochloride and 7.4 equivalents of DIEA were dissolved in anhydrous DCM and added to the resin. The combination was stirred at RT for

30 min, followed by the addition of 100 µL of MeOH and an additional 15 min of stirring. The solution was then drained, and the resin was washed with DMF.

Load Fmoc-Gly-OH to Sieber Amide resin
49 mg of resin (0.03 mmol, 1 eq) was transferred into a 10 mL SPPS vessel. The resin was allowed to swell in anhydrous DMF for 30 min before being drained. Subsequently, the Fmoc group was removed as described in the standard protocol. A mixture of 3 equivalents of Fmoc-Gly-OH, 3 equivalents of PyOxim, and 6 equivalents of DIEA were dissolved in 0.75 mL NMP, allowed to be activated for 5 min, and then added to the resin. After stirring at RT for 30 min, the resin was drained and washed with DMF.

Deprotection of the Fmoc protecting group
Following each coupling, the resin was washed with DMF (x5), and then the Fmoc group was removed using 1 mL of a solution containing 1% HOBt (w/v), 2% DBU (v/v) in DMF, repeated three times. Finally, the resin was washed again with DMF (x4) and then NMP (x1) before the next coupling reaction.

Standard Amino Acid Couplings
Following Fmoc deprotection and subsequent washing steps, amino acid coupling solutions were added to the free amine on the growing peptide chain. The coupling solutions contained 3 equivalents of the appropriate Fmoc-amino acid, 3 equivalents of PyOxim, and 6 equivalents of DIEA in 0.75 mL of NMP. Prior to being added to the resin, the coupling solutions were allowed to sit for 5-10 min for activation. The resin was stirred with the coupling solution for 30 min at RT before being drained and washed.

Peptide building blocks Couplings
Whenever possible and appropriate, dipeptide building blocks (Fmoc-Pro-Hyp(tBu)-OH; Fmoc-Gly-Pro-OH) and the tripeptide building block Fmoc-Gly-Pro-Hyp(tBu)-OH were used while synthesizing each CMP. These building blocks were coupled to the peptide in the same manner as the single amino acids, with the exception that 2 equivalents of building blocks were used in the reaction: The coupling solution contained 2 equivalents of Fmoc peptide building blocks, 2 equivalents of PyOxim, and 4 equivalents of DIEA in 0.5 mL of NMP.

N-terminal modification
Acetylation: Following the removal of the final Fmoc group and the DMF washing, 1 mL of an Ac_2O, DIEA, and DMF (v/v = 1:2:3) was added to the resin and stirred at room temperature for 30 min.
Succinylation: Following the removal of the final Fmoc group and the DMF washing, a solution containing 10 eq of succinic anhydride and 20 eq of DIEA in 1 mL NMP was added to the resin and stirred at room temperature for 30 min.

Cleavage of the protected peptide
Upon completing all appropriate couplings, the resin was washed with 5x DMF and 5x DCM and then air-dried. For 2-chlorotrityl chloride resin, a 3 mL cleavage cocktail comprising 3%

TFA, 2% TIPS, and 95% DCM was added to the resin. In the case of Sieber amide resin, 2% TFA in DCM was used instead. The mixture was stirred for 30 min before being collected and concentrated under reduced pressure. The peptide was then co-evaporated with toluene twice to remove the residual TFA, followed by drying *in vacuo* and used in the peptide ligation without further purification.

Peptide fragment coupling

The intermediate **I5** (1 eq), a peptide with N-terminal free amine on resin, was washed with NMP. The intermediate **I6** (1 eq), PyAOP (3 eq), and DIEA (6 eq) were dissolved in 1 mL NMP, activated for 5 min, and added to the resin. The mixture was stirred at 35 °C for 30 min, followed by DMF washing, DCM washing, and peptide cleavage. For another type of design, in the reaction of intermediate **S4** and **S5**, 1.5 eq of PyAOP and 3 eq of DIEA were used instead, and no activation was performed before coupling.

Final coupling and final cleavage

Peptide intermediates **I4** and **I8** were dissolved in 1 mL anhydrous DCM, followed by adding 10 equivalents of DIEA and 5 equivalents of PyAOP. The mixture was allowed to be agitated at 35 °C for 30 min and the DCM was evaporated afterwards. To the residual solid, a 2 mL cleavage cocktail containing 95% TFA, 2.5% TIPS, and 2.5% H_2O was added. The mixture was agitated for 1 hour at RT before being combined with 50 mL cold ether and precipitated the crude peptide. The peptide was collected by centrifugation, resuspended in cold ether, and collected by centrifugation again. The final pellet was then dissolved in 1 mL of water and stored at 4 ˚°C prior to HPLC purification.

Purification

The peptide solutions were purified using preparative reversed-phase HPLC using acetonitrile and water (with 0.1% TFA added) as the mobile phase. During purification, the column was heated at 80 °C to prevent triple helix formation and aid in separation. The absorbance at 214 nm was monitored to determine the fraction collection, and collected fractions were analyzed using MALDI-TOF MS in positive ion mode. Appropriate fractions were combined and repeatedly lyophilized to yield the desired peptide as a white solid. Purity was then checked using analytical HPLC, with the column heated at 80 °C and a 30 min gradient of 10% to 30% acetonitrile.

CIRCULAR DICHROISM

General measurement of peptide unfolding

Approximately 250 μL of each peptide, with a concentration of 200 μM in PBS buffer, was placed into a 1mm quartz cuvette. The ellipticity of these solutions was then measured from 260 to 190 nm while holding the temperature at 4 °C. Measurements were obtained in triplicate and then converted to mean residue ellipticity to generate the CD scan curves for each peptide. Following this, the solutions were heated at a rate of 5 °C/h, starting at 4 °C and ending at 92 °C while monitoring the absorbance at 210, 215, 220, and 225 nm. These measurements, obtained in triplicate, were converted to mean residue ellipticity. The melting temperature for each peptide, T_m, was determined by fitting the data to a two-state model to find the temperature at which 50% of starting ellipticity was lost, as described in the subsequent section.

T_m measurement with the variation of concentration

Each peptide sample was prepared at concentrations of 12.5, 25, 50, 100, 200, and 400 μM in PBS buffer and was placed into 1mm quartz cuvettes for the measurement. The exceptions were the samples with concentrations of 12.5 μM and 25 μM, for which the 1 cm quartz cuvettes were used, given their lower concentration. The solutions were heated at a rate of 5 °C /h, starting at 4 °C and ending at 92 °C while monitoring the absorbance at 210, 215, 220, and 225 nm. Additional measurements were performed on peptide 4 and H-(GPO)$_7$-NH$_2$ with a temperature changing rate of 3 °C /h for both unfolding and refolding experiments. The measured data were converted to mean residue ellipticity and fitted to a two-state model to find the temperature at which 50% of starting ellipticity was lost as T_m.

Hysteresis measurement

250 μL of the peptide at the concentration of 200 μM in PBS buffer was placed into a 1 mm quartz cuvette. The peptide solution was heated at a rate of 1 °C/min starting at 4 °C and ending at 92 °C, then cooled at the same rate from 92 °C to 4 °C, while monitoring the absorbance at 225 nm. The actual heating and cooling rates differed slightly from the instrument setting, calculated from the measurement time. The instrument spent 88 min for heating and 92 min for cooling, resulting in an average temperature changing rate of 61 °C/h.

Kinetics measurement

The peptide folding and unfolding kinetics were monitored at temperatures ranging from 10 °C to 80 °C with 10 °C intervals, using peptide samples at 200 μM concentration in PBS buffer. Prior to the measurement, a 1 mm quartz cuvette was inserted into the spectrometer and preheated or precooled to the desired temperature. When the cuvette reached the target temperature, the peptide solution was promptly injected, and the ellipticity monitoring at 225 nm was immediately started. For peptides that exhibit rapid unfolding within seconds, initiating CD monitoring prior to peptide injection is necessary to ensure more accurate data acquisition. The measurement was performed with a data interval of 2 seconds and a digital integration time of 2 seconds. For the folding experiments, the sample solutions were

preheated to 80 °C for 5 min to unfold the peptide, and for the unfolding experiments, the peptides were allowed to equilibrate at room temperature for 20 min. The apparent rate constants were obtained by fitting the acquired folding and unfolding kinetic data to the model of first-order reversible reaction (Eq. 1). Experiments with high temperature changes result in a longer time for thermal equilibration, and the initial period of data was not used for fitting.

$$\theta(t) = Ae^{-k_{app}t} + B \quad (1)$$

Isothermal kinetics measurements were conducted to determine the folding/unfolding rate constant under various urea concentrations. The peptide solution was mixed with urea solution to achieve the target urea concentration, and the peptide folding or unfolding was monitored by CD signal at 25 °C.

DIFFERENTIAL SCANNING CALORIMETRY

DSC measurement of peptide unfolding

Approximately 600 µL of each peptide, with a concentration of 200 µM in PBS buffer, was loaded into the sample cell (active cell volume = 300 µL) while the PBS buffer was loaded in the reference cell. The measurements were performed with the heating rate of 5 °C/h, starting at 3 °C and ending at 92 °C, under the constant pressure of 3 atm. The blank data was measured with PBS buffer in both sample and reference cells.

The melting temperature for each peptide, T_m, and the folding ΔH and ΔS were determined by fitting the data to a two-state model discussed below.

SIZE EXCLUSION CHROMATOGRAPHY

Samples were diluted to 100 µM in ultrapure water from a Mill-Q system (Millipore Sigma). Strands were denatured by heating to 60 ºC for 15 mins, then re-annealed by cooling at 4 ºC for 24 hours. Samples were injected (2 to 4 µL) onto a Waters Acquity UPLC Protein BEH SEC Column (125 Å, 1.7 µm, 4.6 mm x 150 mm) connected to a Waters Acquity Ultra Performance LC liquid chromatography mass spectrometry (LCMS) system. Samples were run at 0.3 mL/min for 10 mins at either room temperature (approx. 25 ºC) or 40 ºC. Running buffer consisted of 1 mM ammonium acetate in ultrapure Milli-Q water adjusted to pH 6 with glacial acetic acid. Masses of each compound were measured by electrospray ionization (ESI) from 100 to 2000 g/mol range. Spectra were analyzed with the MestReNova software suite.

THERMODYNAMIC MODEL OF Bis β-Turn CMP FOLDING

CD data fitting of trimeric CMPs (H-(GPO)$_7$-NH$_2$, H-(GPO)$_7$-NH$_2$ and H-(GPO)$_7$-NH$_2$) was based on previously published thermodynamics model and method[3].

Similar to the method fitting the CD data of trimeric collagen mimetic peptides, we postulated that the CD ellipticity has a linear dependence on temperature for the folded and unfolded species, as described in Eq. (9). For Bis β-turn CMP, the thermodynamic model is a simpler monomeric reversible reaction U ⇌ F, in which U and F denote the unfolded peptide and the folded triple helical peptide, respectively.

$$\mathrm{U} \rightleftharpoons \mathrm{F} \tag{2}$$

$$\Delta G = \Delta H° - T\Delta S° = -RT \ln K \tag{3}$$

$$K = \frac{[F]}{[U]} = \frac{F_{eq}}{1-F_{eq}} \tag{4}$$

$$\Delta G(T_m) = -RT_m \ln\left(\frac{0.5}{0.5}\right) = \Delta H° - T_m\Delta S° = 0 \tag{5}$$

$$\Delta S° = \frac{\Delta H°}{T_m} \tag{6}$$

$$-RT \ln\left(\frac{F_{eq}}{1-F_{eq}}\right) = \Delta H°\left(1 - \frac{T}{T_m}\right) \tag{7}$$

$$F_{eq} = \frac{1}{1+e^{\frac{\Delta H°}{RT}\left(1-\frac{T}{T_m}\right)}} \tag{8}$$

$$\theta(T) = \left(E_f + D_f \cdot T\right)F_{eq} + \left(E_u + D_u \cdot T\right)\left(1 - F_{eq}\right) \tag{9}$$

Assuming Δ*H*° and Δ*S*° are not temperature dependent, we could derive the equilibrium folding fraction $F_{eq}(T)$ in Eq. (8). Combining equations (8) and (9) results in a relationship between ellipticity θ and temperature *T* which could be directly measured as CD melting curve. The six parameters, Δ*H*°, T_m, E_f, D_f, E_u, and D_u, could be determined by non-linear fitting of the θ-*T* data set. The Δ*S*° could be obtained from Eq. (6).

$$\Delta H = \Delta H(T_m) + \int_{T_m}^{T} \Delta C_p(T')dT' \tag{10}$$

$$\Delta S = \Delta S(T_m) + \int_{T_m}^{T} \frac{\Delta C_p(T')}{T'}dT' \tag{11}$$

$$C_{p,obs}(T) = C_{p,base}(T) + C_{p,trans}(T) \tag{12}$$

$$C_{p,base}(T) = C_{p,f}F_{eq} + C_{p,u}\left(1 - F_{eq}\right) \tag{13}$$

$$C_{p,f} = E_f + D_f \cdot T \quad (14)$$

$$C_{p,u} = E_u + D_u \cdot T \quad (15)$$

$$C_{p,trans}(T) = \Delta H \frac{dF_{eq}}{dT} = \left[\Delta H(T_m) + (E_u - E_f)(T - T_m) + \frac{1}{2}(D_u - D_f)(T - T_m)^2\right] \frac{dF_{eq}}{dT} \quad (16)$$

The processing of the DSC data generally followed the previously published method,[4] with the collagen folding model altered from trimeric to monomeric. The observed heat capacity is the sum of the peptide heat capacity $C_{p,base}$ and the heat capacity of peptide unfolding $C_{p,trans}$ (Eq. 12). The baseline can be fitted in the same way as CD (Eq. 13-15), resulting in the heat capacity of the folded and unfolded peptides $C_{p,f}$ and $C_{p,u}$. The heat capacity of the folding-unfolding transition can be derived from the same thermodynamic model as CD, but the ΔH and ΔS are temperature dependent, derived from the heat capacities from the baseline fitting (Eq. 10, 11, and 16). DSC data fitting also gives a peptide concentration c_{fit} based on the heat capacity, which is consistent with the prepared sample concentration c_{sample} based on the UV-Vis measurement.

All non-linear curve fitting in this work was performed by SciPy (scipy.optimize.leastsq).[5]

DISCUSSION OF THE KINETICS AND MECHANISMS OF Bis β-Turn CMP FOLDING

The proposed two-step kinetics model for fitting k_{app}-T data.

$$U \underset{k_{-1}}{\overset{k_1}{\rightleftharpoons}} I \underset{k_{-2}}{\overset{k_2}{\rightleftharpoons}} F$$

Applying steady-state approximation:

$$\frac{dI}{dt} = 0$$

$$\frac{dU}{dt} = \frac{-k_1 k_2 U + k_{-1}k_{-2}(1-U)}{k_{-1} + k_2}$$

Integration:

$$U(t) = \frac{1}{K+1} + Ce^{-\frac{k_1 k_2 + k_{-1}k_{-2}}{k_{-1}+k_2}t}$$

$$k_{app} = \frac{k_1 k_2 + k_{-1}k_{-2}}{k_{-1} + k_2} = \frac{k_1 k_{-2}(K+1)}{k_1 + k_{-2}K}$$

$$k_f = \frac{k_1 k_2}{k_{-1} + k_2} = \frac{k_1 k_{-2}}{\frac{k_1}{K} + k_{-2}}; k_u = \frac{k_{-1}k_{-2}}{k_{-1} + k_2} = \frac{k_1 k_{-2}}{k_1 + k_{-2}K}$$

Assuming k_1 and k_2 follow Arrhenius' law:

$$\ln k_1 = A_1 - \frac{E_{a,1}}{RT} \; ; \; \ln k_{-2} = A_{-2} - \frac{E_{a,-2}}{RT}$$

The fitting was performed with the input k_{app} - T data to determine the four parameters A_1. $E_{a,1}$, A_{-2}, $E_{a,-2}$.

PEPTIDE ANALYTICAL HPLC

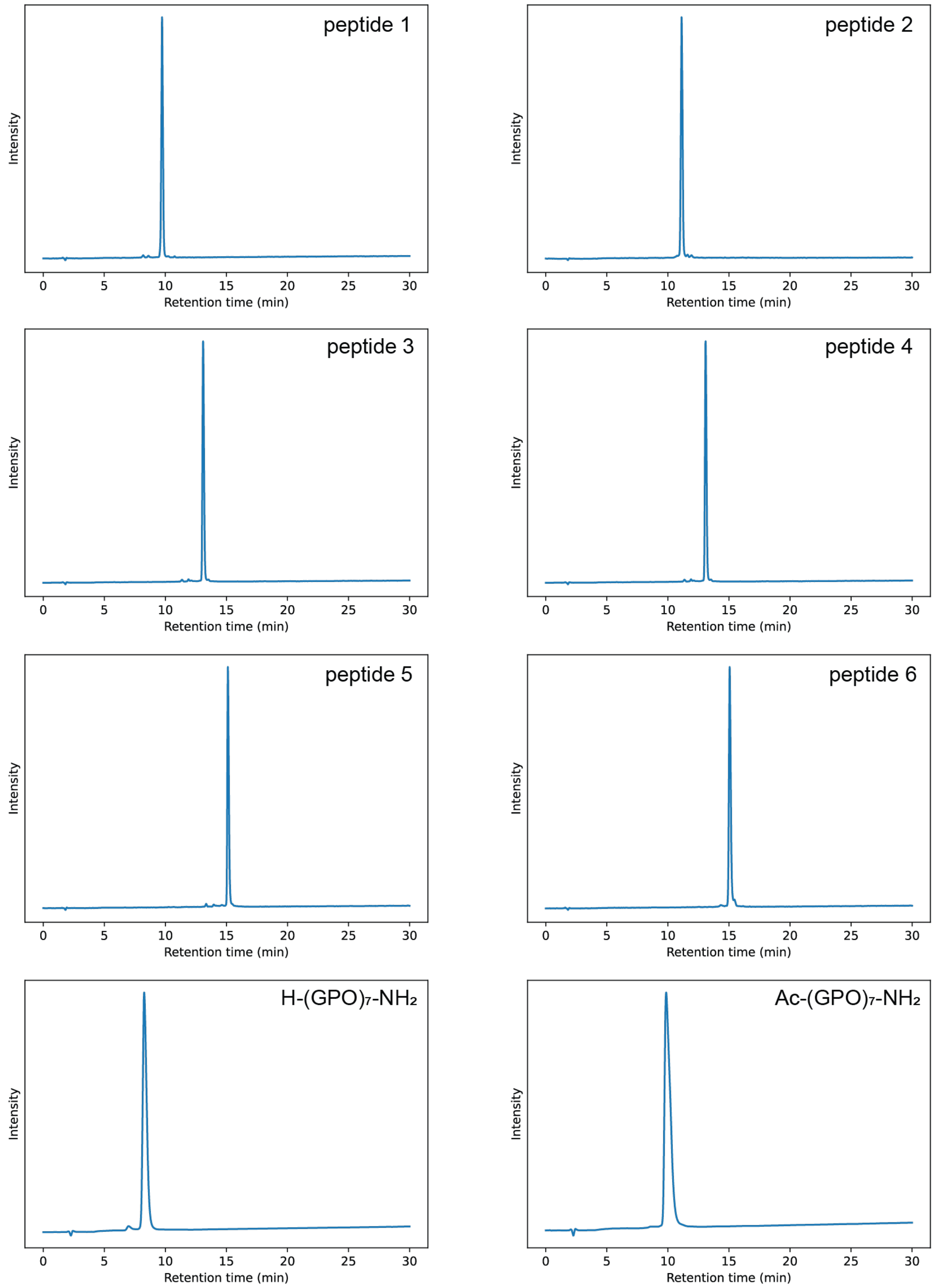

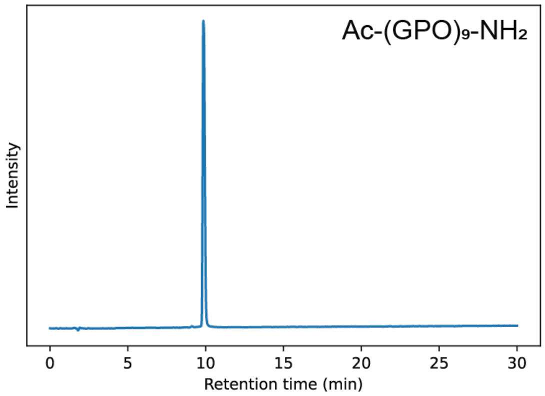

MALDI-TOF MS

Peptide	MW calc	$[M+H]^+$	$[M+Na]^+$	m/z found
Peptide 1	2185.01	2186.02	2208.00	2208.51
Peptide 2	2452.13	2453.14	2475.12	2475.28
Peptide 3	2986.37	2987.38	3009.36	3009.94
Peptide 4	3253.50	3254.51	3276.49	3276.83
Peptide 5	3787.74	3788.75	3810.73	3811.69
Peptide 6	4054.86	4055.87	4077.85	4078.49
H-(GPO)$_7$-NH$_2$	1886.88	1887.89	1909.87	1888.15
Ac-(GPO)$_7$-NH$_2$	1928.89	1929.90	1951.88	1952.18
Ac-(GPO)$_9$-NH$_2$	2463.13	2464.138	2486.12	2486.67

Peptide Folding Stability Characterization
Peptide 1

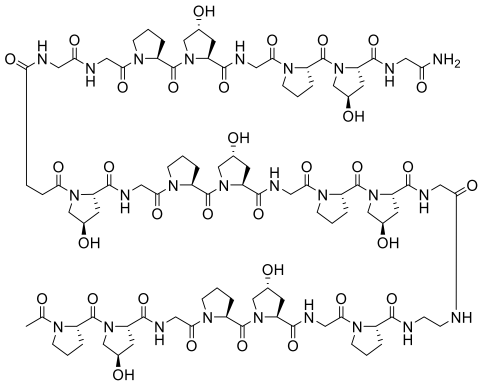

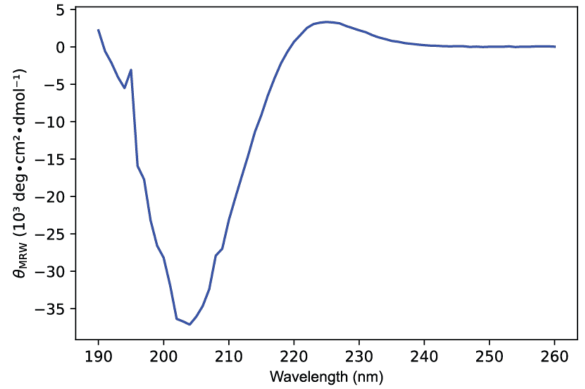

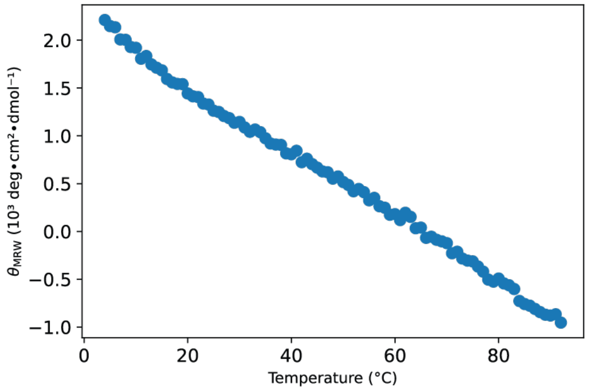

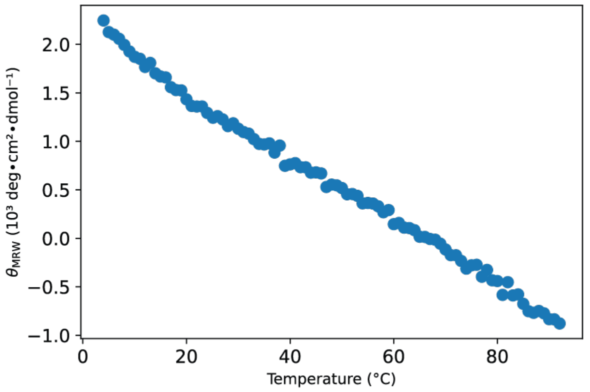

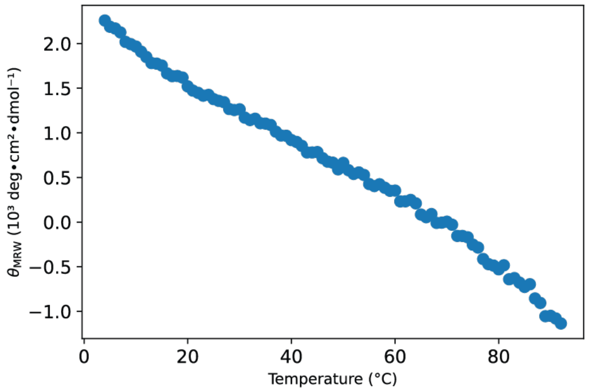

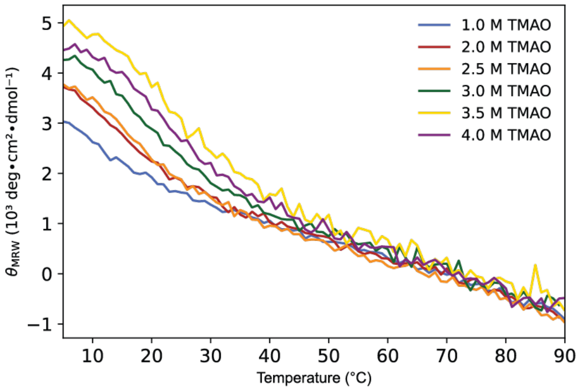

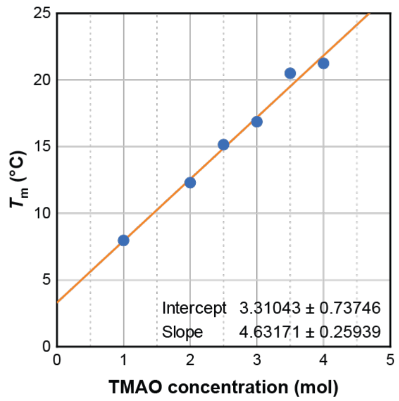

Peptide 2

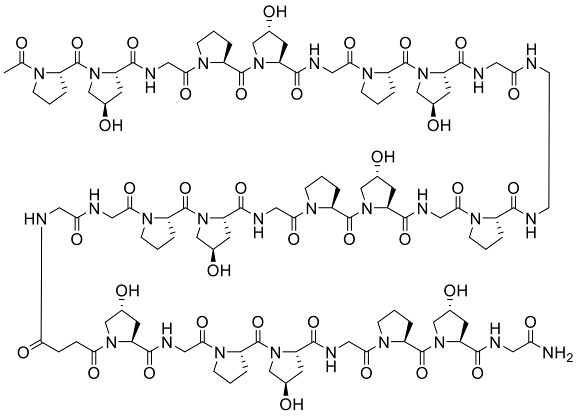
OH
OH
NH₂

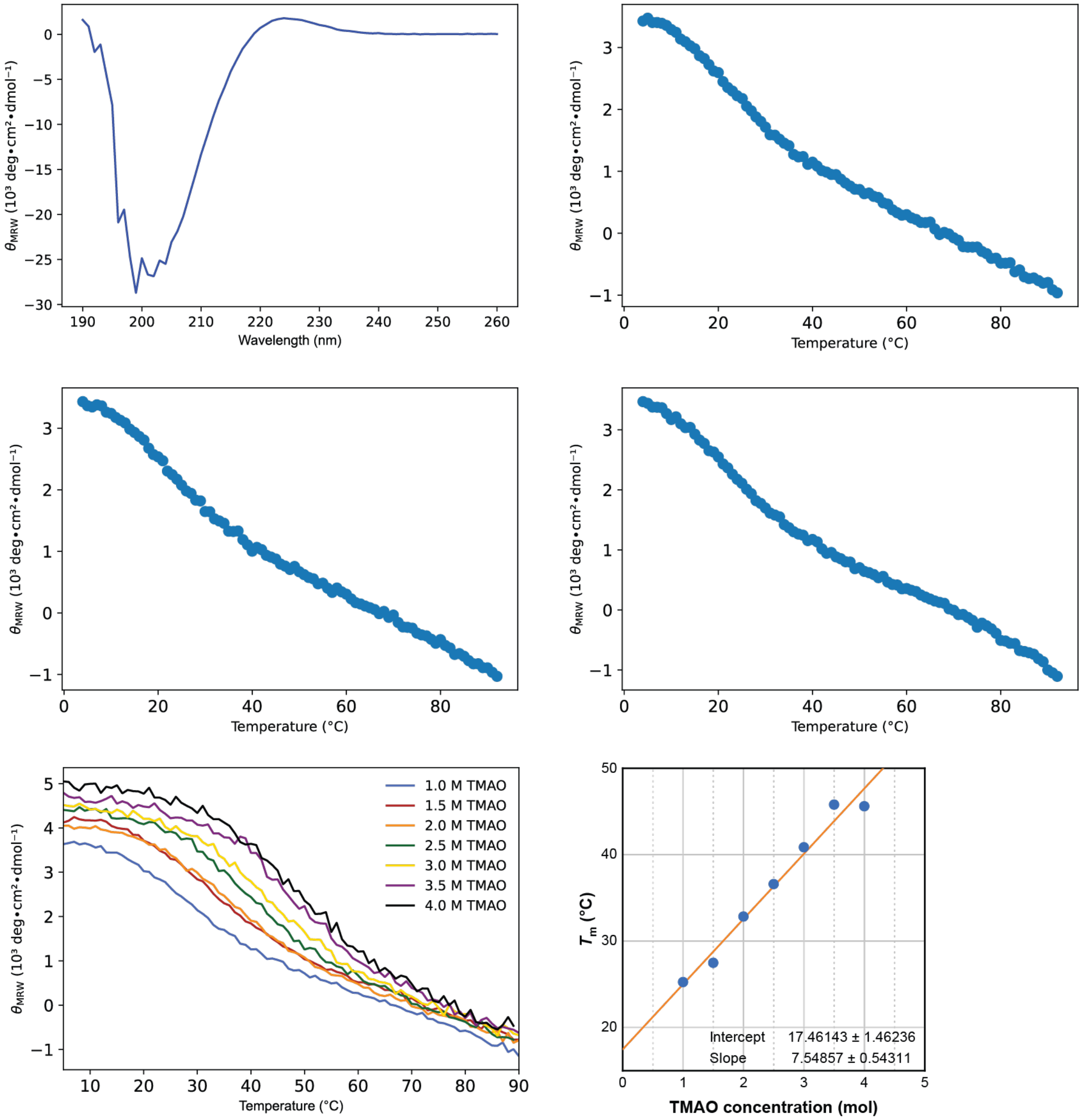
θ_{MRW} (10³ deg•cm²•dmol⁻¹)
Wavelength (nm)
Temperature (°C)
1.0 M TMAO
1.5 M TMAO
2.0 M TMAO
2.5 M TMAO
3.0 M TMAO
3.5 M TMAO
4.0 M TMAO
T_m (°C)
TMAO concentration (mol)
Intercept 17.46143 ± 1.46236
Slope 7.54857 ± 0.54311

Peptide 3

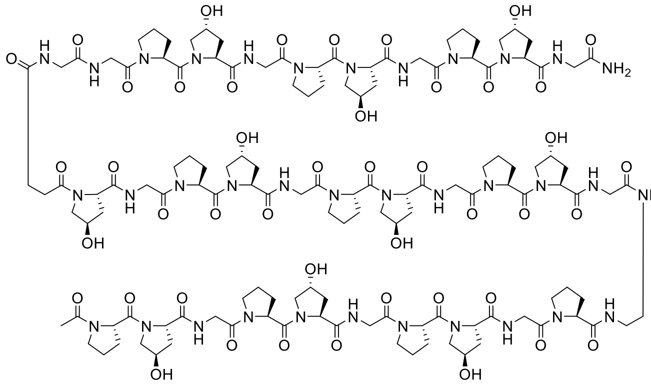
OH
NH2
NH

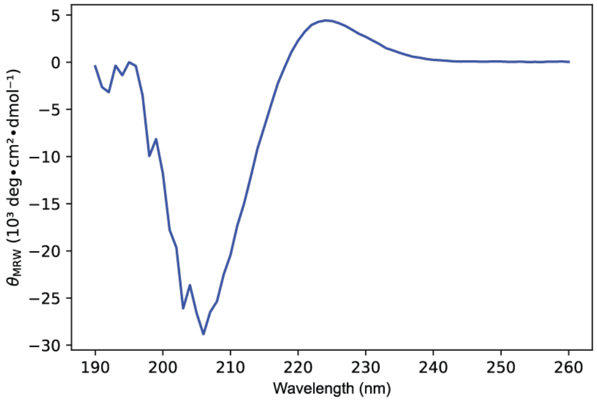
θ_{MRW} (10^3 deg•cm²•dmol⁻¹)
Wavelength (nm)

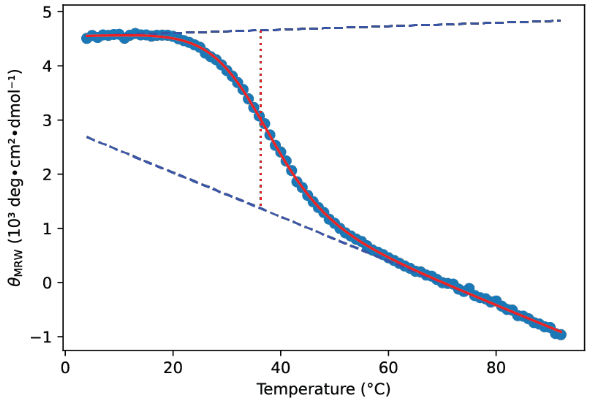
θ_{MRW} (10^3 deg•cm²•dmol⁻¹)
Temperature (°C)

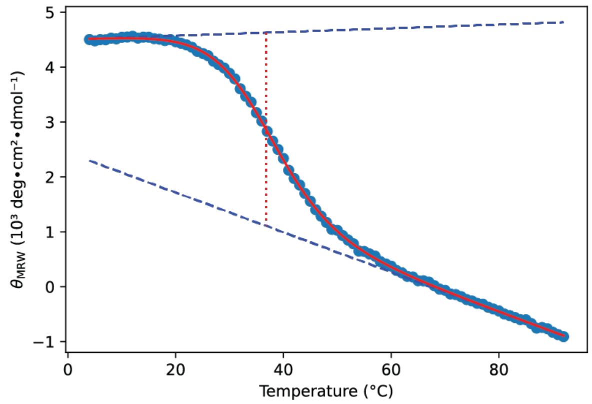
θ_{MRW} (10^3 deg•cm²•dmol⁻¹)
Temperature (°C)

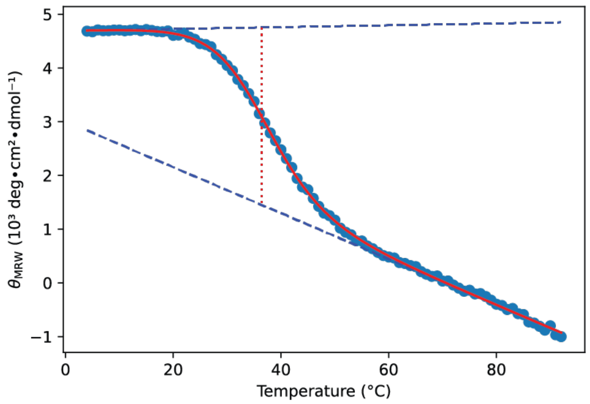
θ_{MRW} (10^3 deg•cm²•dmol⁻¹)
Temperature (°C)

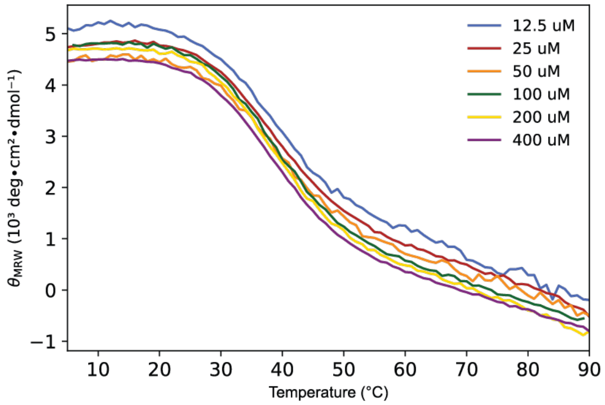
12.5 uM
25 uM
50 uM
100 uM
200 uM
400 uM
θ_{MRW} (10^3 deg•cm²•dmol⁻¹)
Temperature (°C)

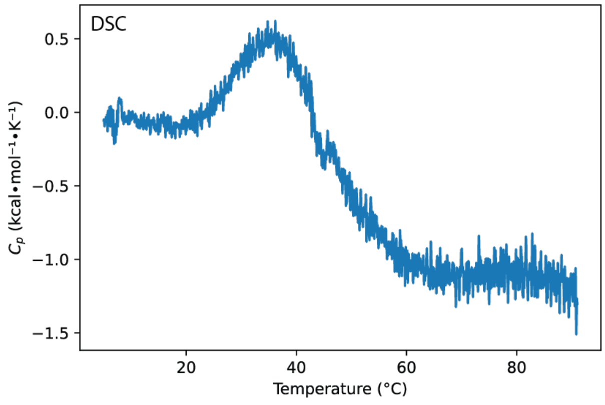
DSC
C_p (kcal•mol⁻¹•K⁻¹)
Temperature (°C)

Peptide 4

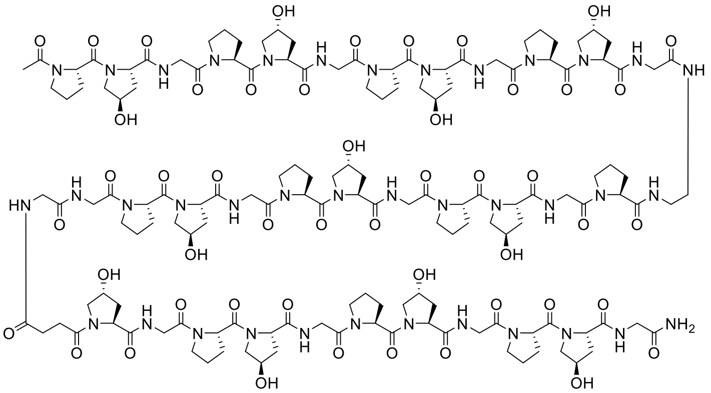

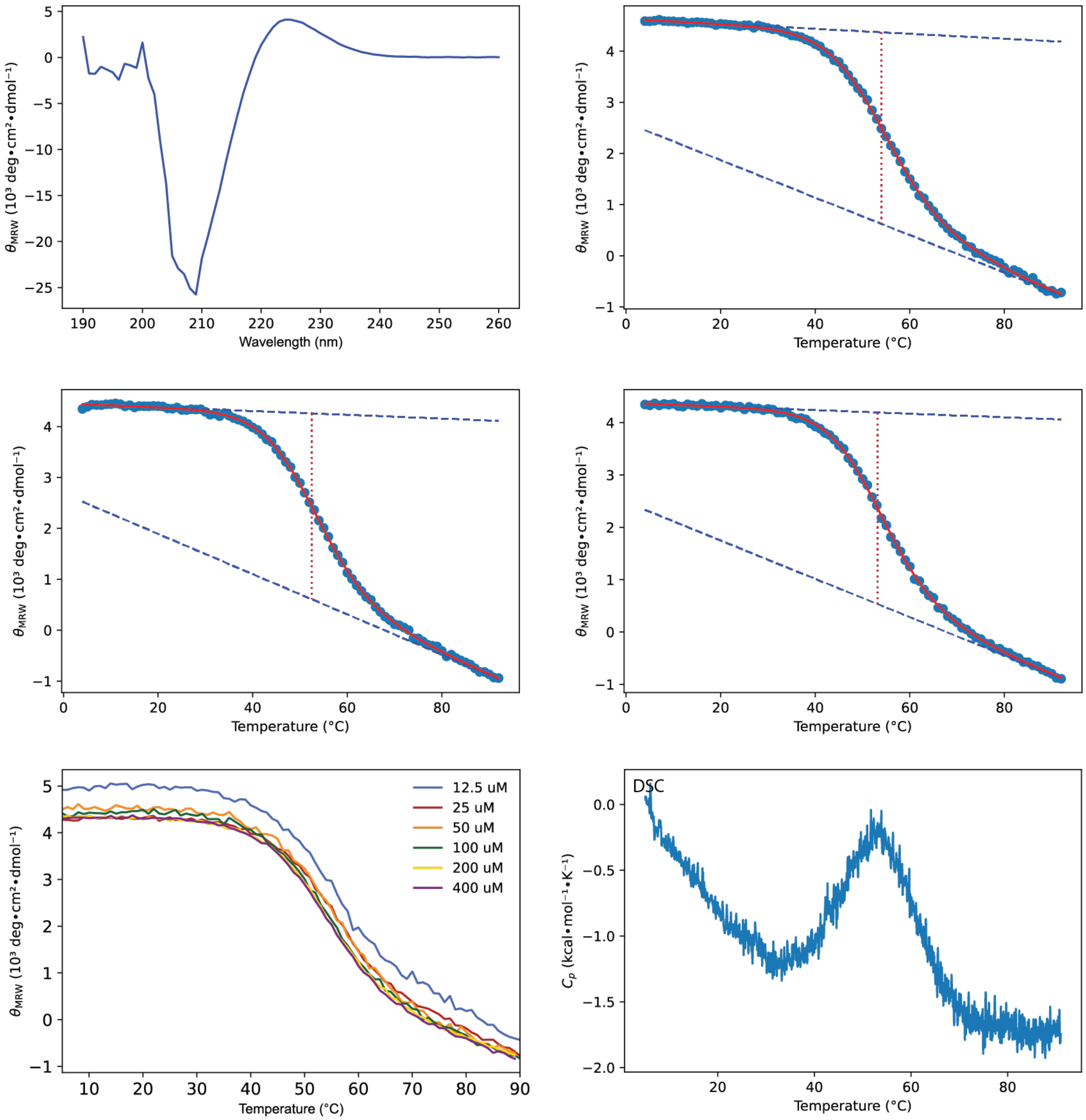

θ_{MRW} (10³ deg•cm²•dmol⁻¹)
Wavelength (nm)
Temperature (°C)
12.5 uM
25 uM
50 uM
100 uM
200 uM
400 uM
DSC
C_p (kcal•mol⁻¹•K⁻¹)

Peptide 5

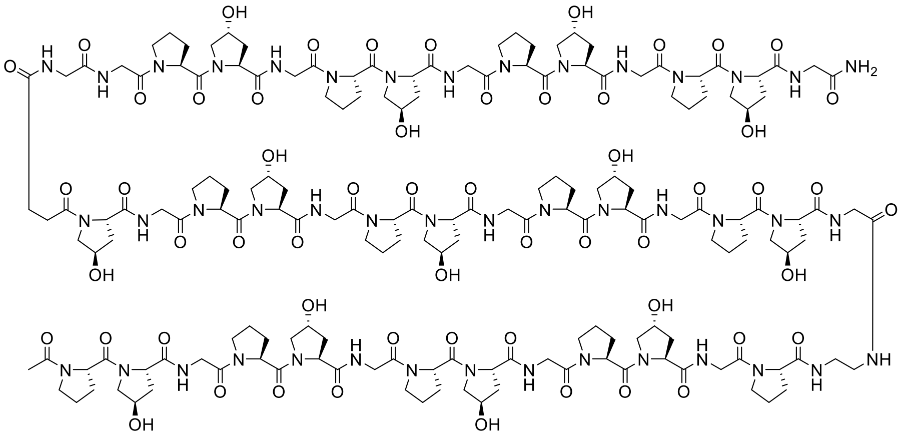

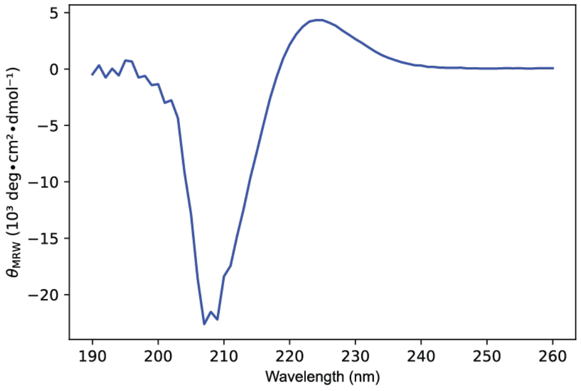

θ_{MRW} (10^3 deg•cm²•dmol⁻¹)
Wavelength (nm)

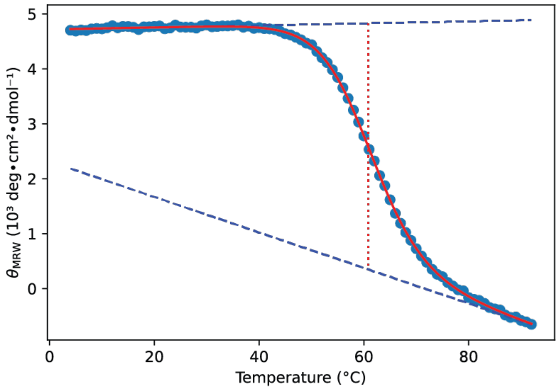

θ_{MRW} (10^3 deg•cm²•dmol⁻¹)
Temperature (°C)

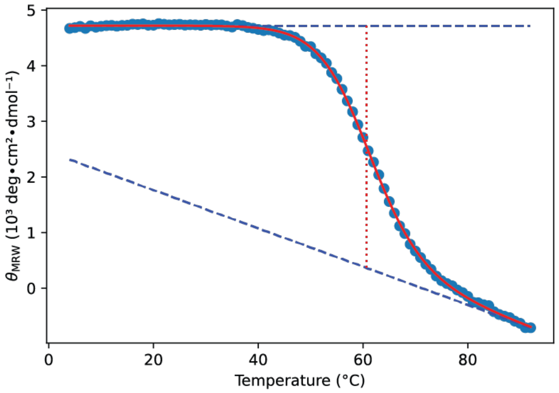

θ_{MRW} (10^3 deg•cm²•dmol⁻¹)
Temperature (°C)

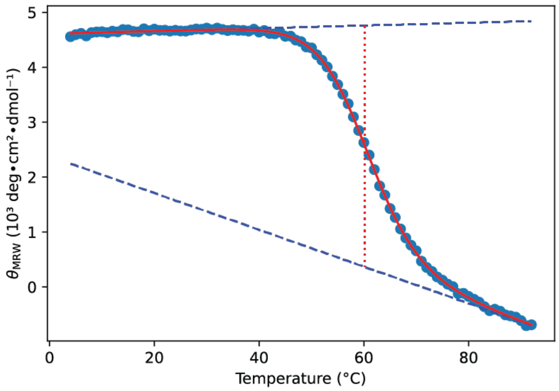

θ_{MRW} (10^3 deg•cm²•dmol⁻¹)
Temperature (°C)

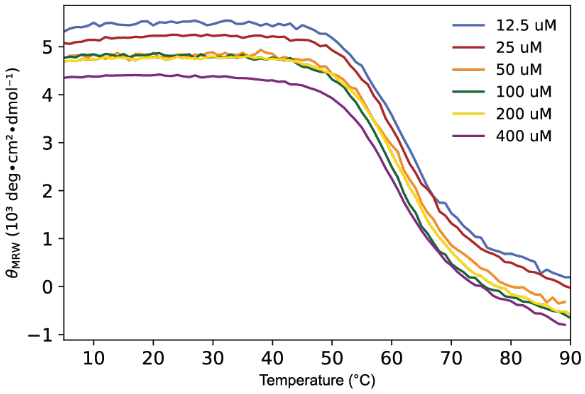

12.5 uM
25 uM
50 uM
100 uM
200 uM
400 uM
θ_{MRW} (10^3 deg•cm²•dmol⁻¹)
Temperature (°C)

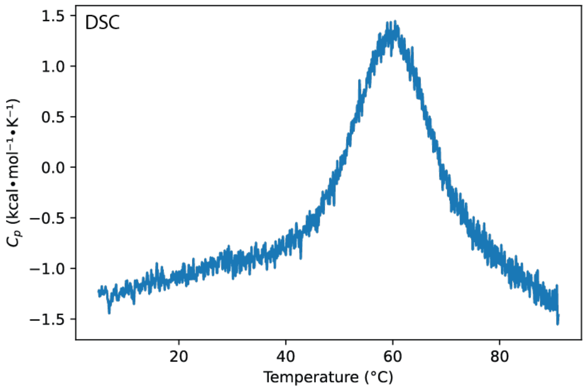

DSC
C_p (kcal•mol⁻¹•K⁻¹)
Temperature (°C)

Peptide 6

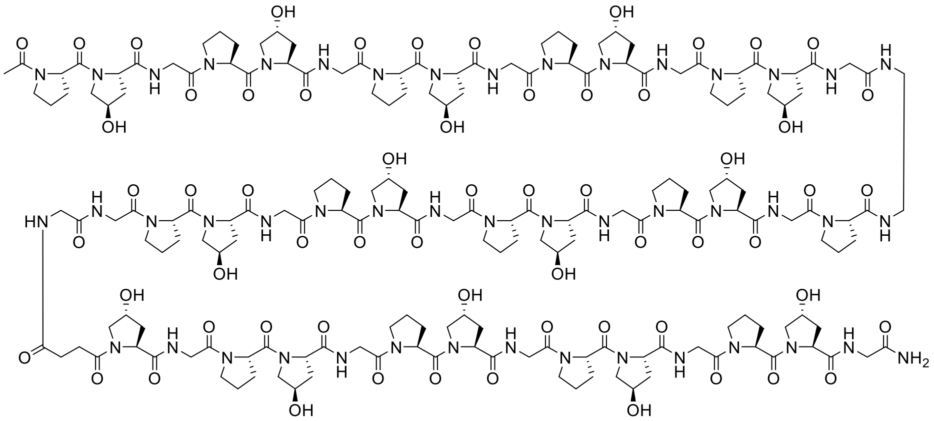

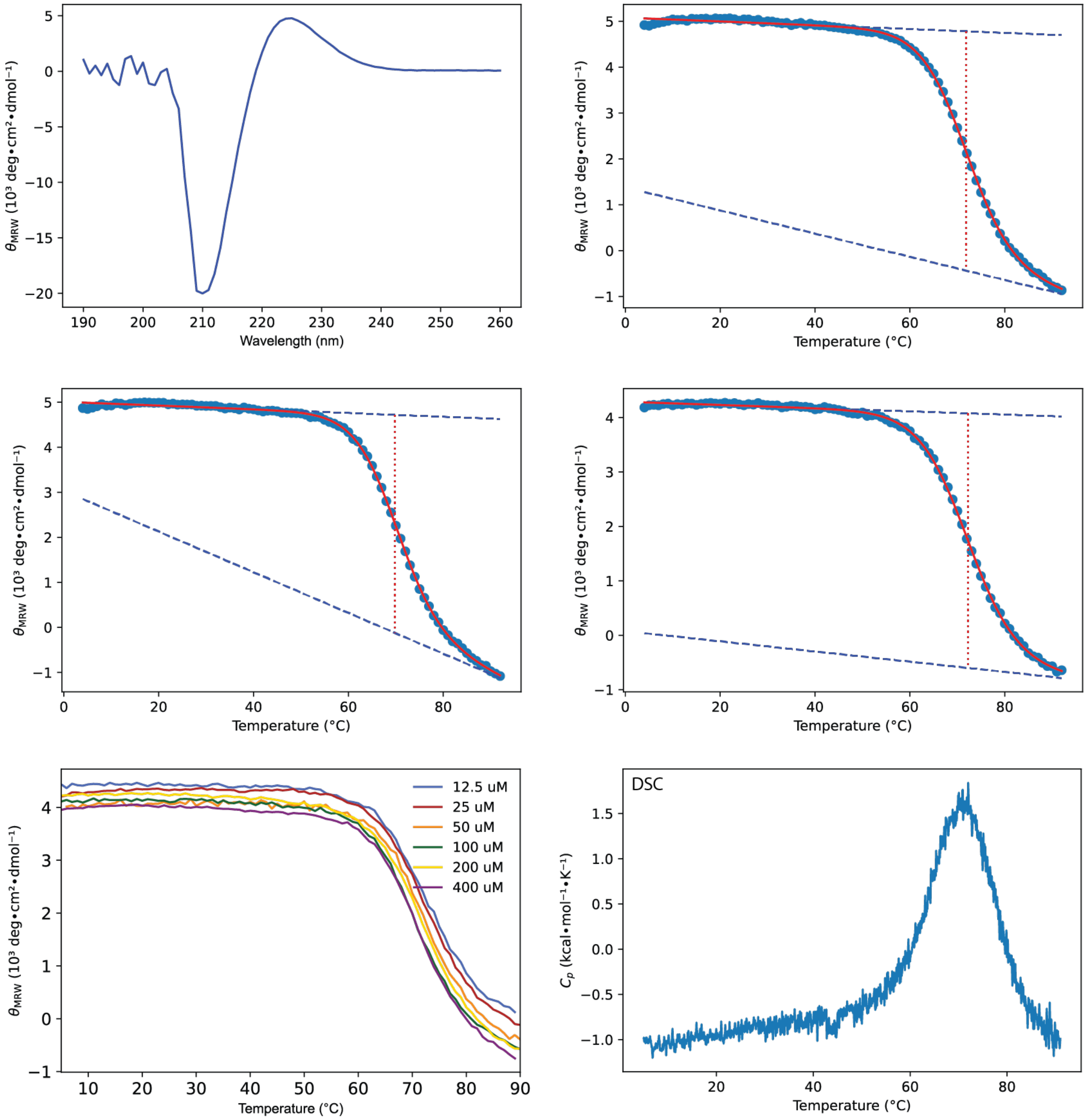
θMRW (10³ deg•cm²•dmol⁻¹)
Wavelength (nm)
Temperature (°C)
12.5 uM
25 uM
50 uM
100 uM
200 uM
400 uM
DSC
Cp (kcal•mol⁻¹•K⁻¹)

H-(GPO)$_7$-NH$_2$

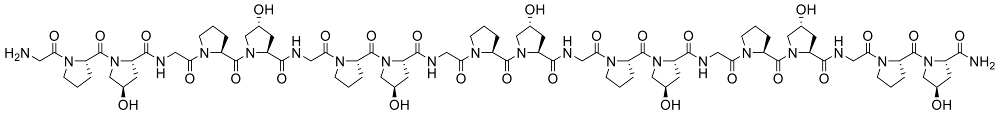

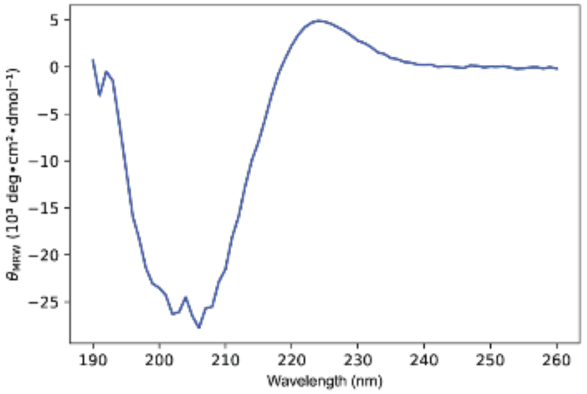

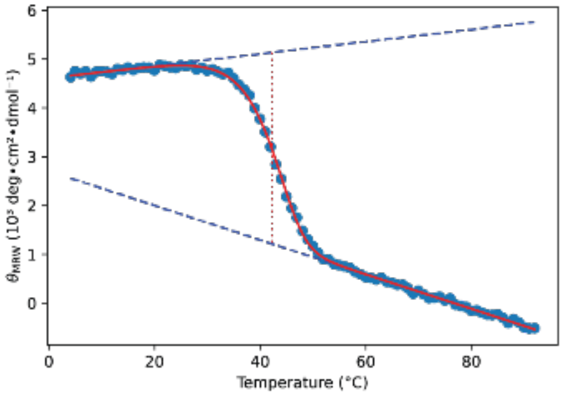

Ac-(GPO)$_7$-NH$_2$

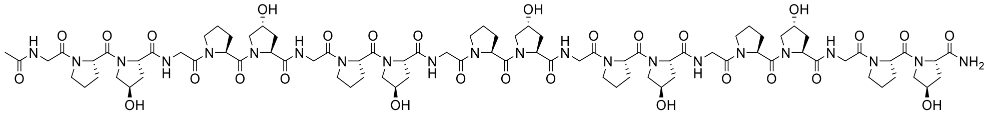

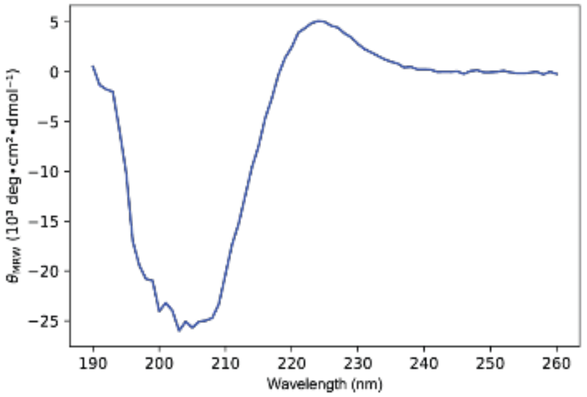

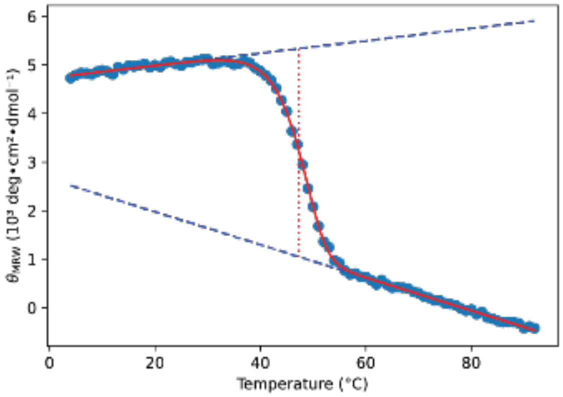

Ac-(GPO)$_9$-NH$_2$

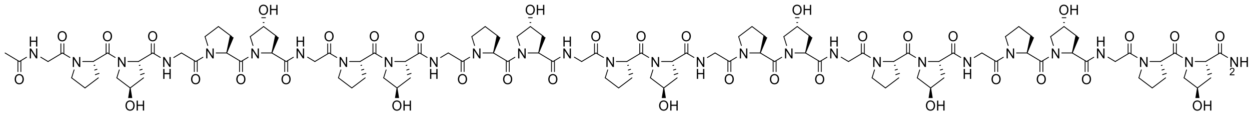

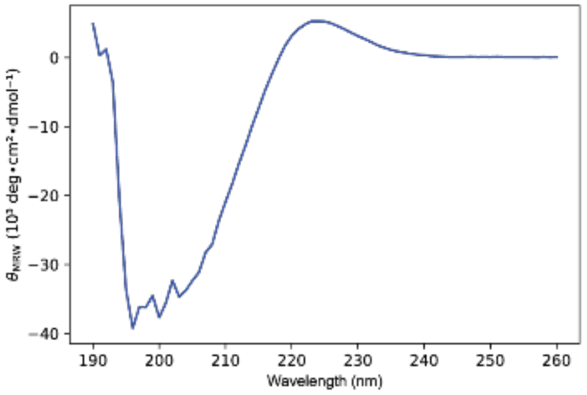

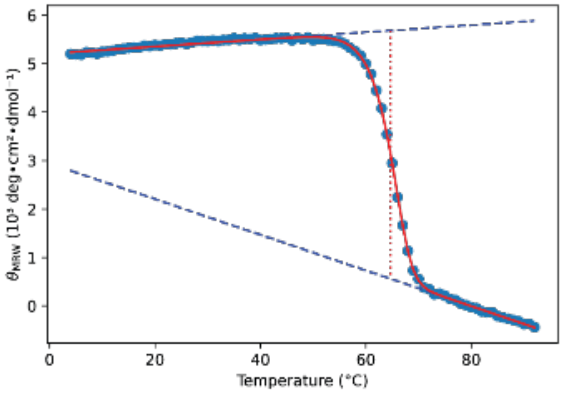

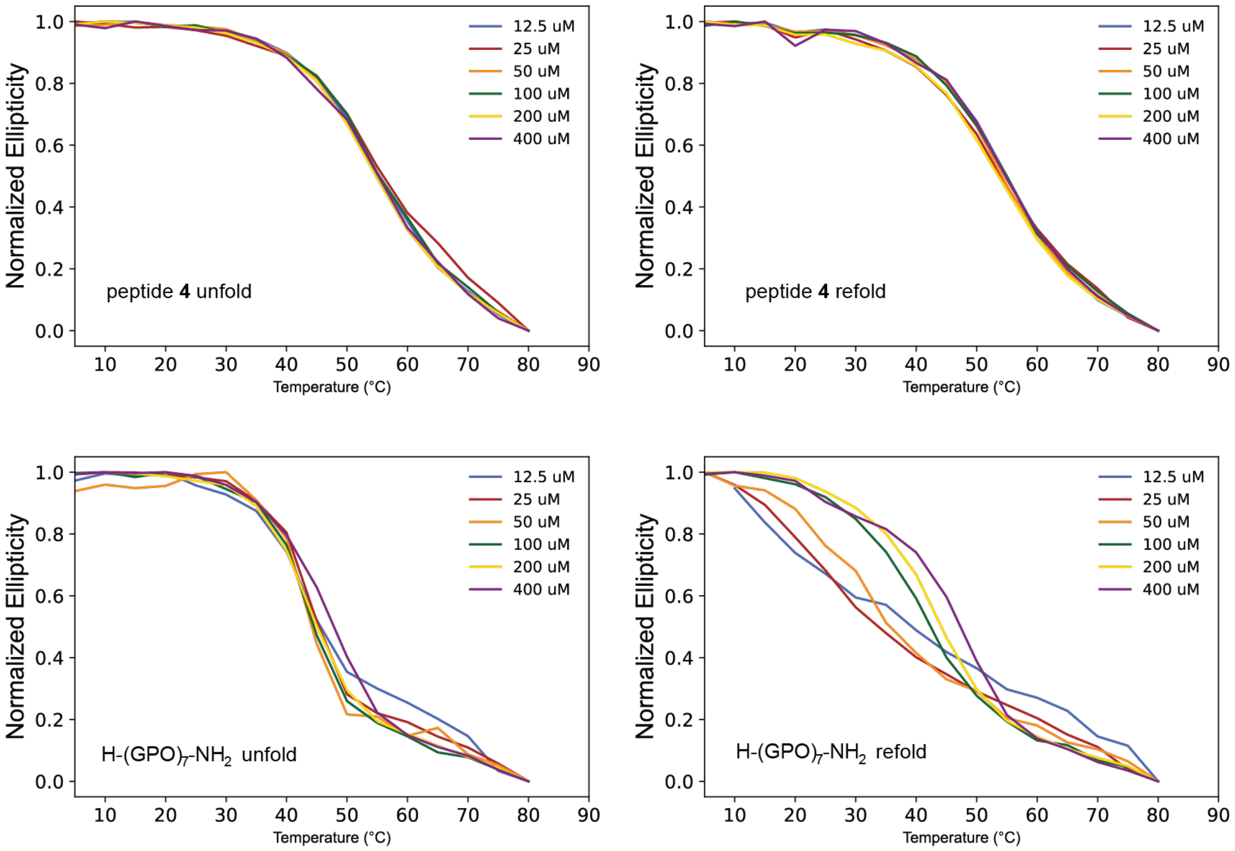

Normalized Ellipticity
Temperature (°C)
12.5 uM
25 uM
50 uM
100 uM
200 uM
400 uM
peptide 4 unfold
peptide 4 refold
H-(GPO)7-NH2 unfold
H-(GPO)7-NH2 refold

Kinetics Measurement
Peptide 3

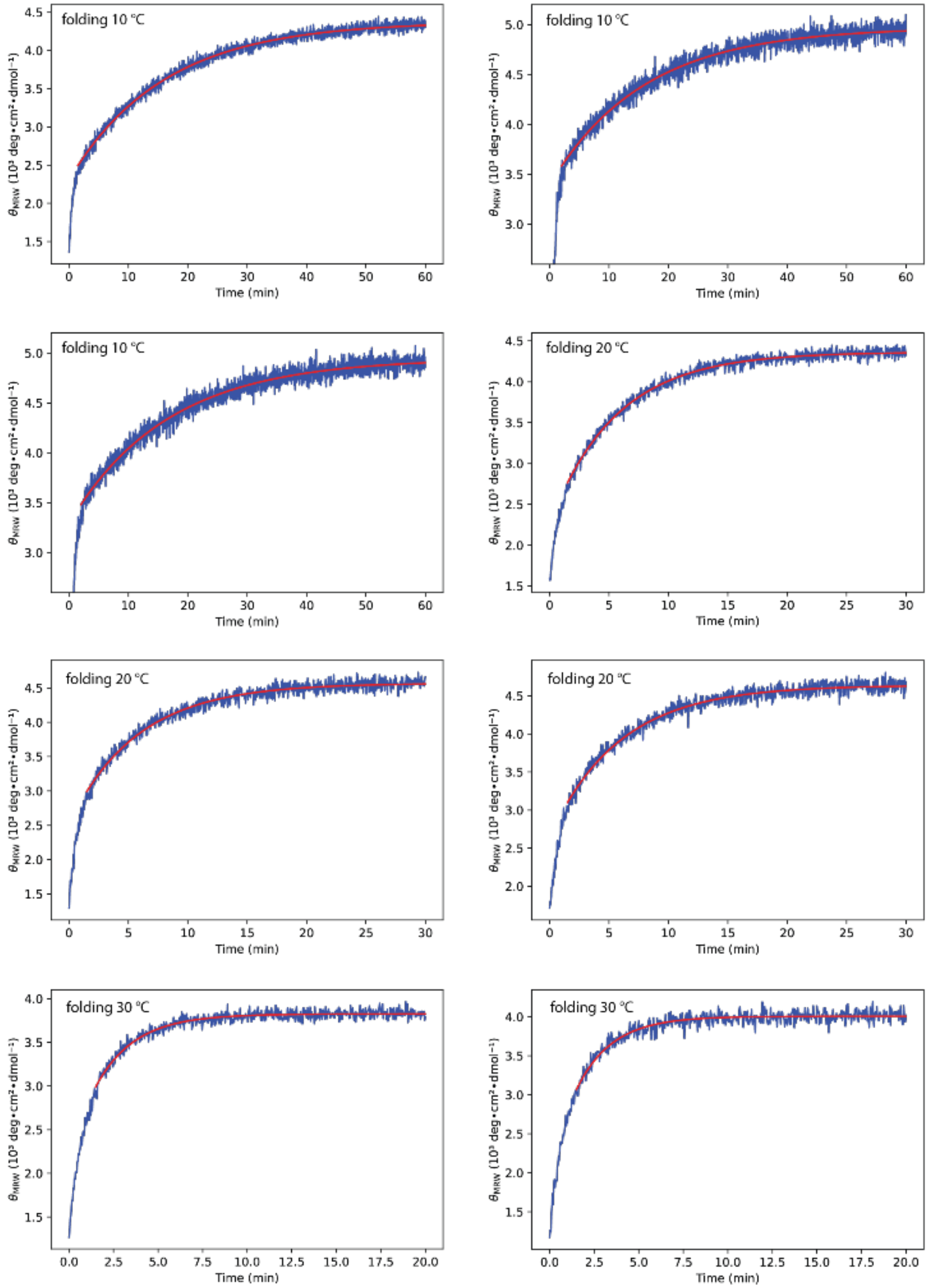

Peptide 3

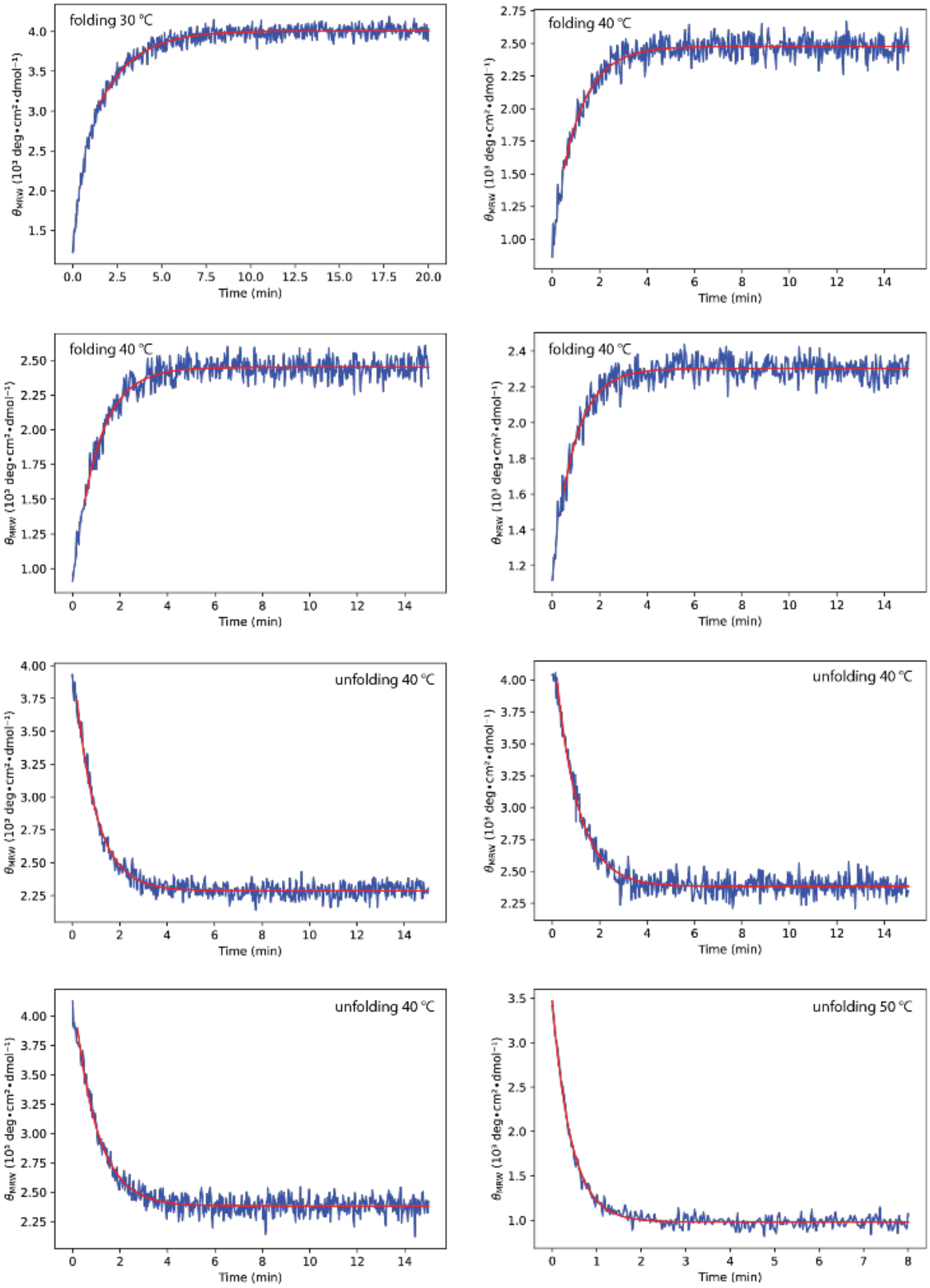
folding 30 ℃
folding 40 ℃
folding 40 ℃
folding 40 ℃
unfolding 40 ℃
unfolding 40 ℃
unfolding 40 ℃
unfolding 50 ℃
θ_{MRW} (10³ deg•cm²•dmol⁻¹)
Time (min)

Peptide 3

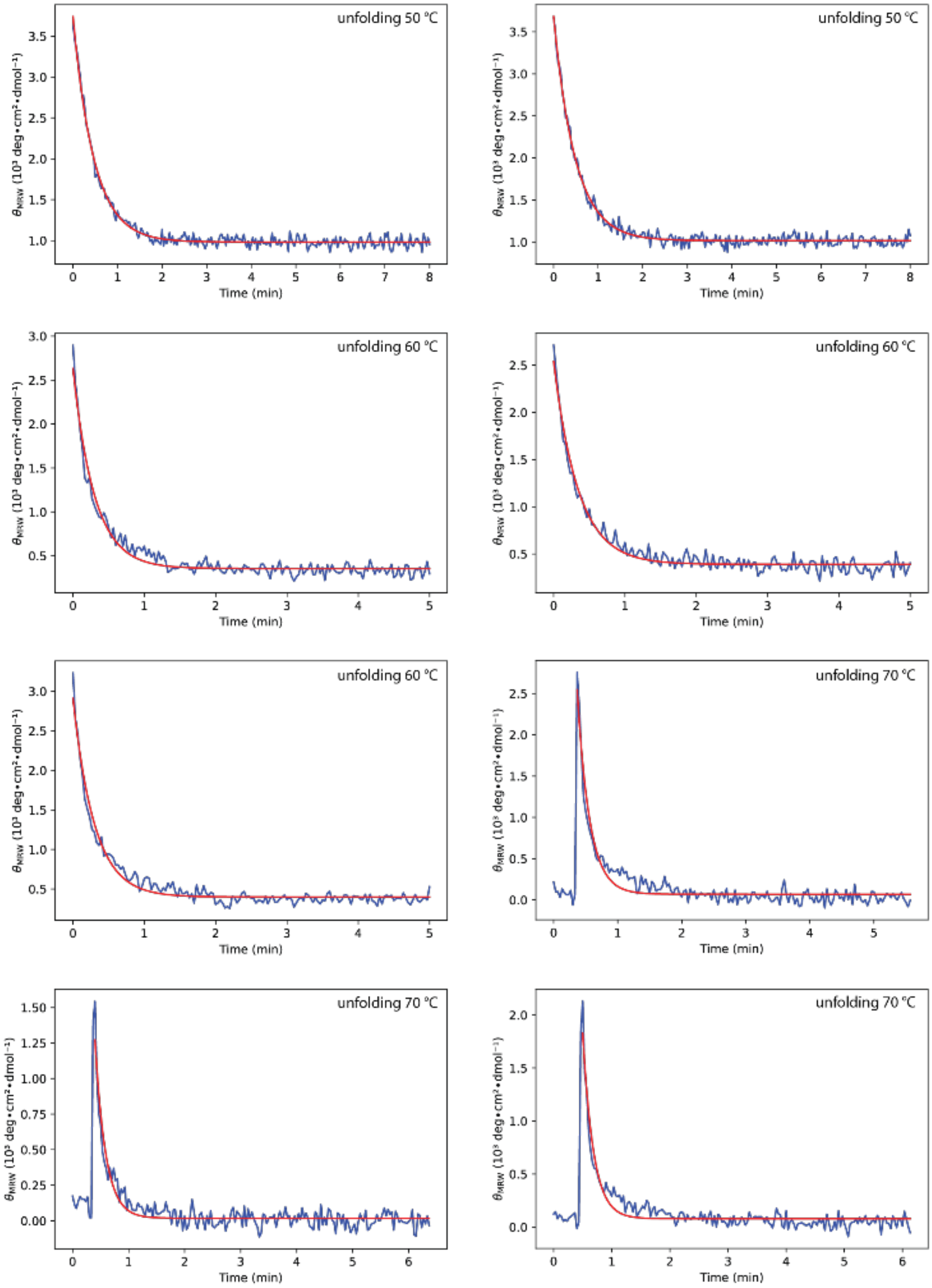
unfolding 50 °C
unfolding 50 °C
unfolding 60 °C
unfolding 60 °C
unfolding 60 °C
unfolding 70 °C
unfolding 70 °C
unfolding 70 °C
θ_{MRW} (10³ deg•cm²•dmol⁻¹)
Time (min)

Peptide 3

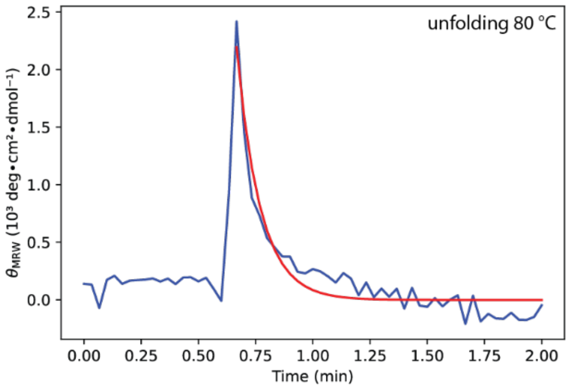
unfolding 80 °C
θ_{MRW} (10³ deg•cm²•dmol⁻¹)
Time (min)

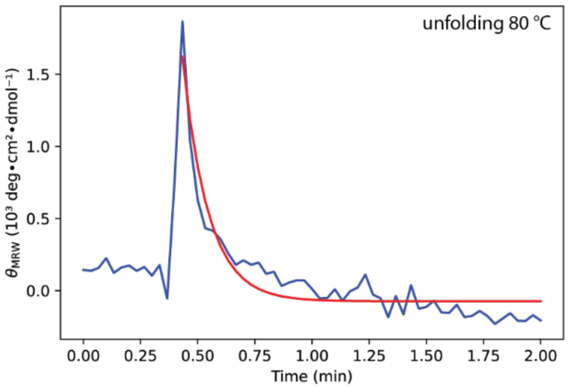
unfolding 80 °C
θ_{MRW} (10³ deg•cm²•dmol⁻¹)
Time (min)

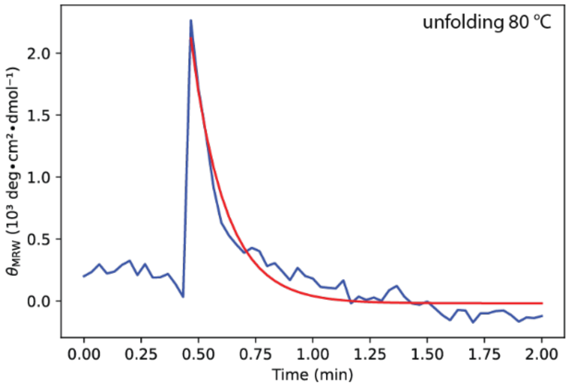
unfolding 80 °C
θ_{MRW} (10³ deg•cm²•dmol⁻¹)
Time (min)

Peptide 4

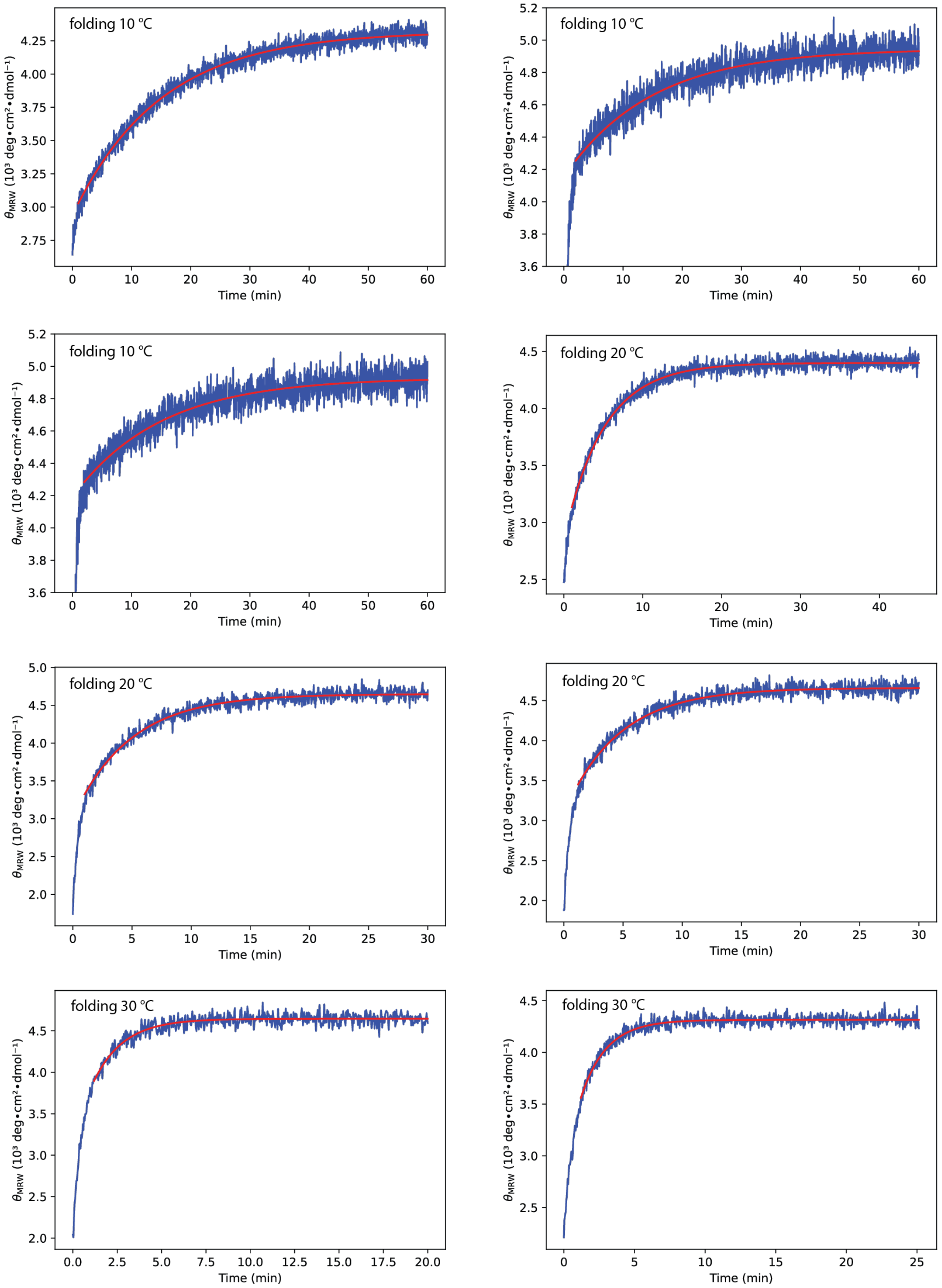

folding 10 °C
folding 10 °C
folding 10 °C
folding 20 °C
folding 20 °C
folding 20 °C
folding 30 °C
folding 30 °C
θ_{MRW} (10³ deg•cm²•dmol⁻¹)
Time (min)

Peptide 4

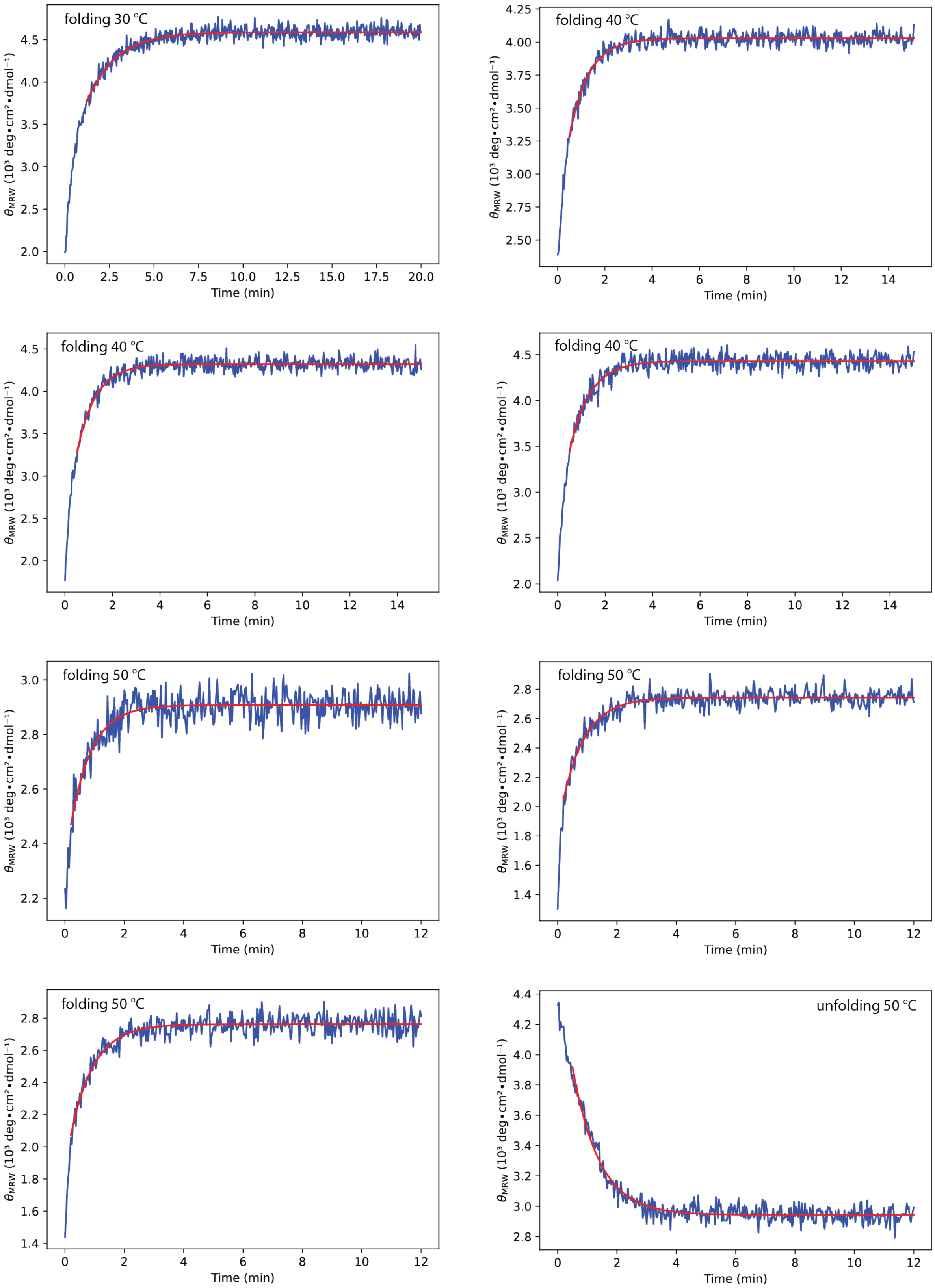
folding 30 °C
folding 40 °C
folding 40 °C
folding 40 °C
folding 50 °C
folding 50 °C
folding 50 °C
unfolding 50 °C
θ_{MRW} (10³ deg•cm²•dmol⁻¹)
Time (min)

Peptide 4

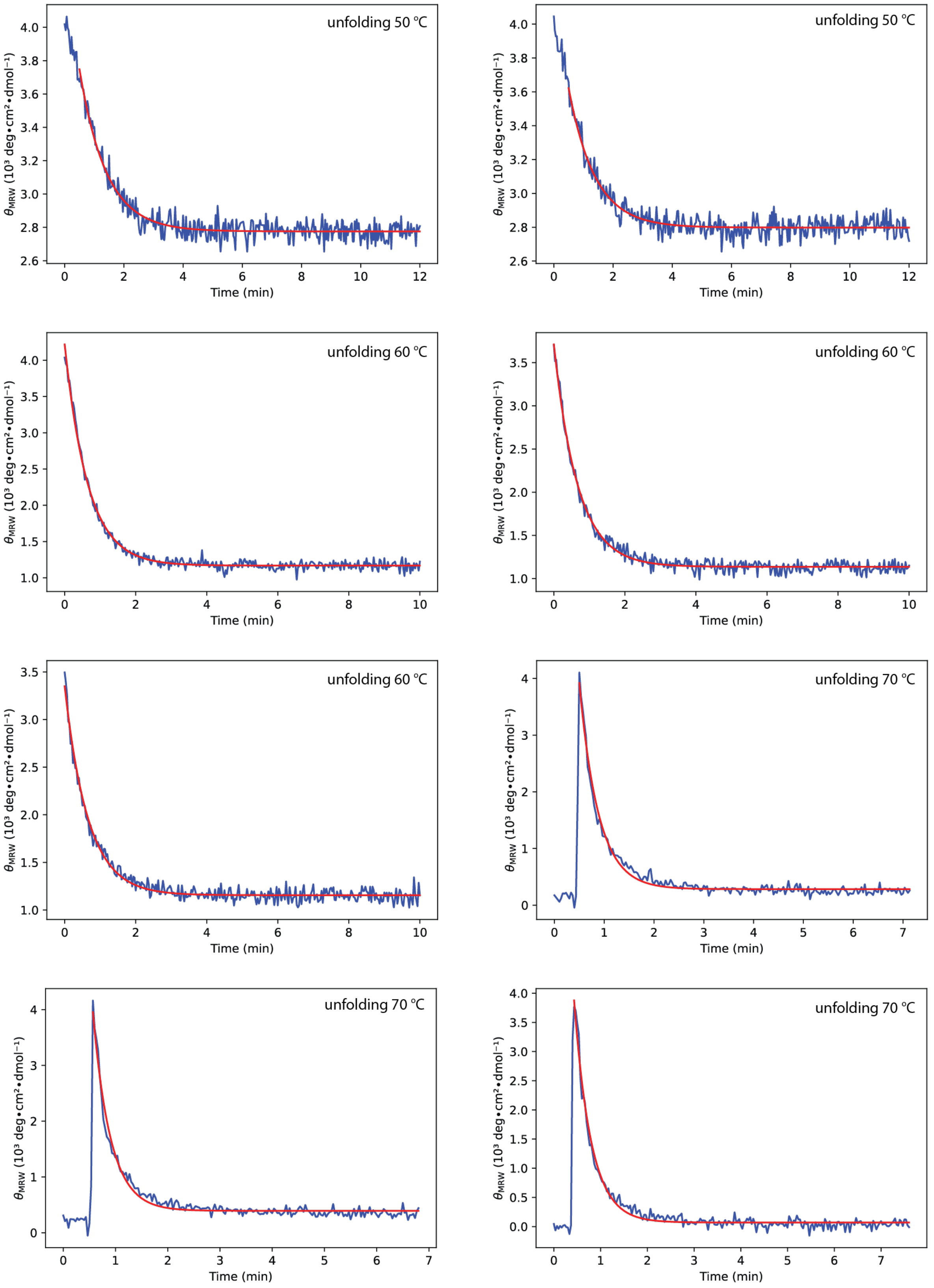
unfolding 50 ℃
unfolding 50 ℃
unfolding 60 ℃
unfolding 60 ℃
unfolding 60 ℃
unfolding 70 ℃
unfolding 70 ℃
unfolding 70 ℃
θ_{MRW} (10³ deg•cm²•dmol⁻¹)
Time (min)

Peptide 4

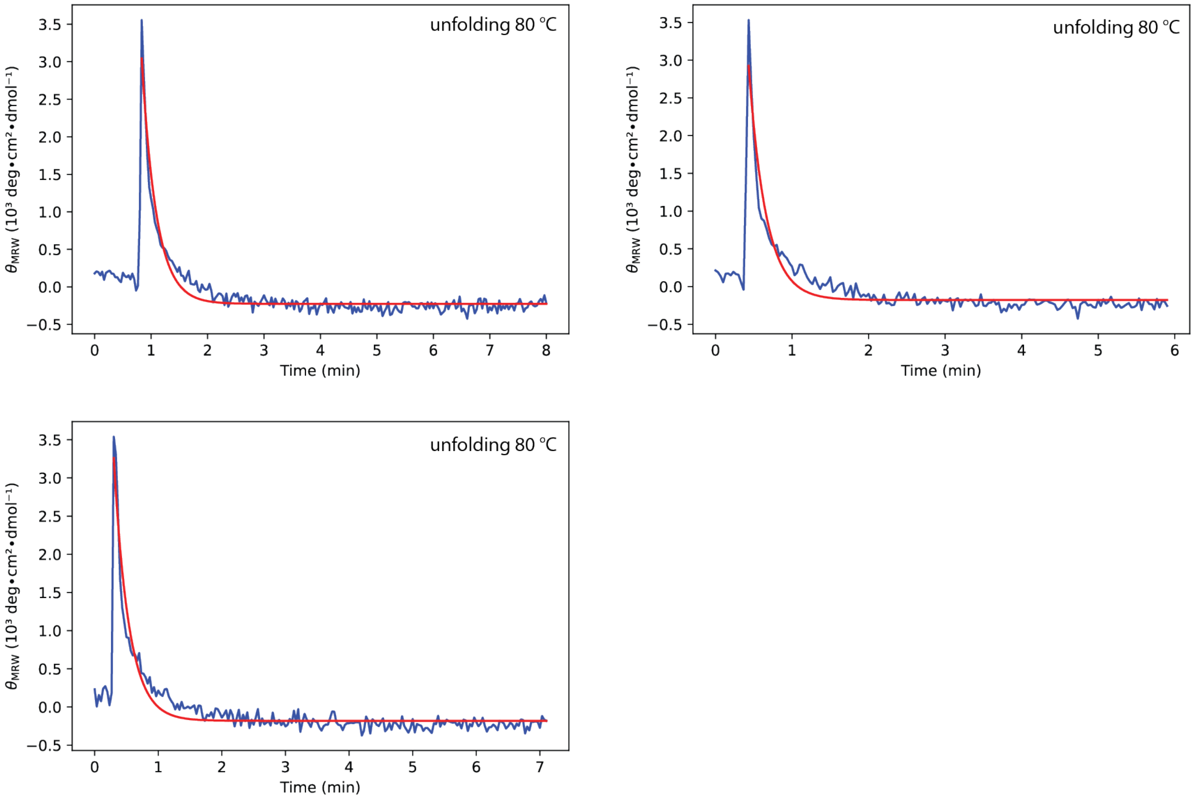
unfolding 80 °C
unfolding 80 °C
unfolding 80 °C
θ_{MRW} (10³ deg•cm²•dmol⁻¹)
Time (min)

Peptide 5

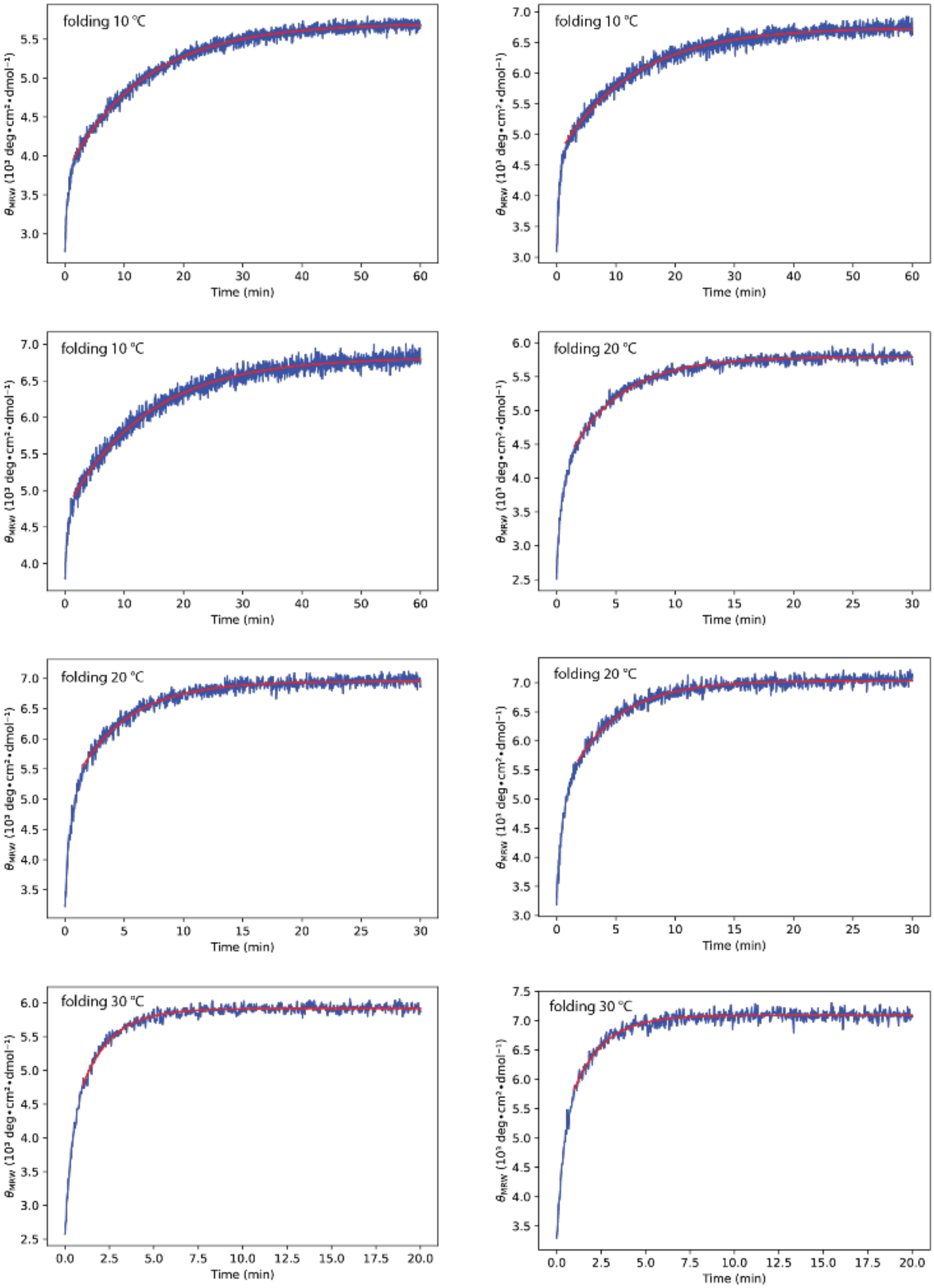

folding 10 °C
folding 10 °C
folding 10 °C
folding 20 °C
folding 20 °C
folding 20 °C
folding 30 °C
folding 30 °C
θ_{MRW} (10^3 deg•cm^2•dmol^{-1})
Time (min)

Peptide 5

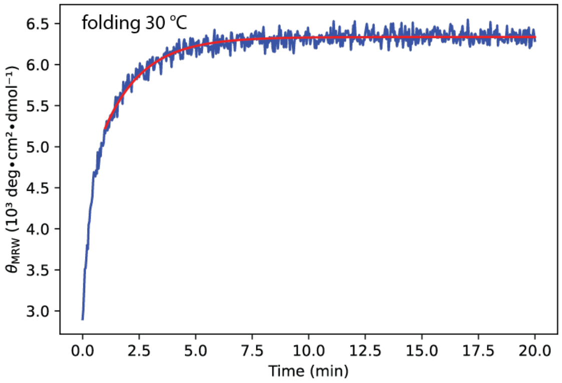

folding 30 °C
θ_{MRW} (10^3 deg•cm^2•dmol^{-1})
Time (min)

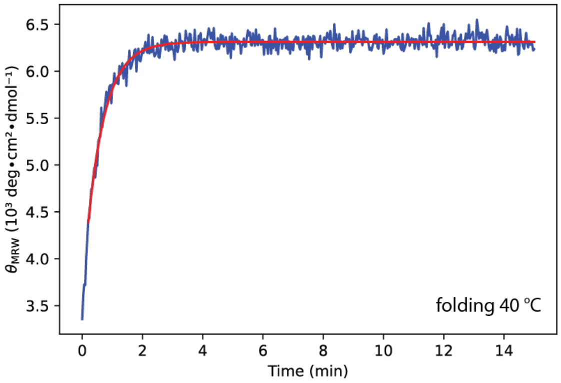

folding 40 °C
θ_{MRW} (10^3 deg•cm^2•dmol^{-1})
Time (min)

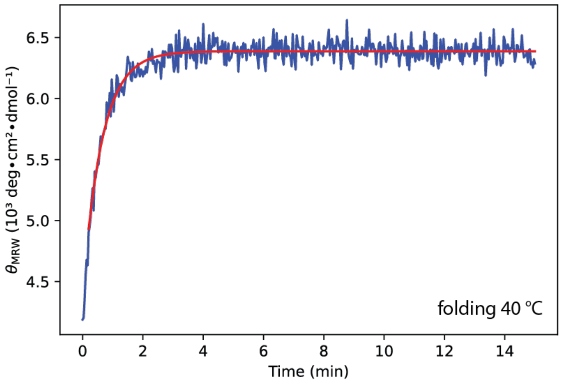

folding 40 °C
θ_{MRW} (10^3 deg•cm^2•dmol^{-1})
Time (min)

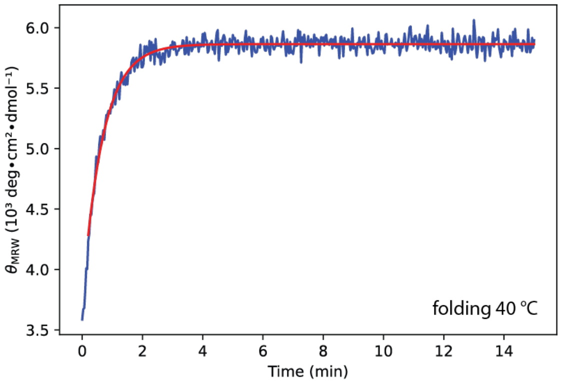

folding 40 °C
θ_{MRW} (10^3 deg•cm^2•dmol^{-1})
Time (min)

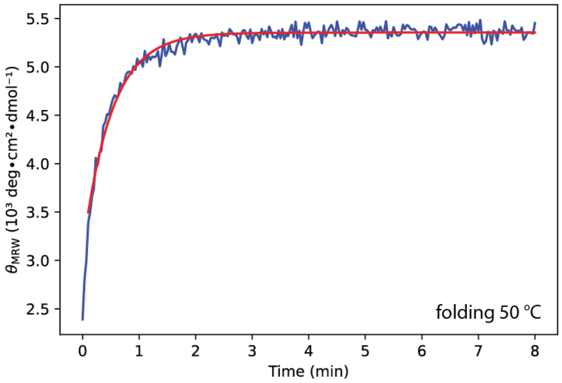

folding 50 °C
θ_{MRW} (10^3 deg•cm^2•dmol^{-1})
Time (min)

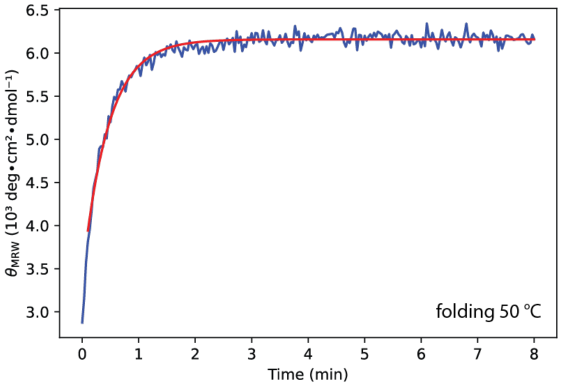

folding 50 °C
θ_{MRW} (10^3 deg•cm^2•dmol^{-1})
Time (min)

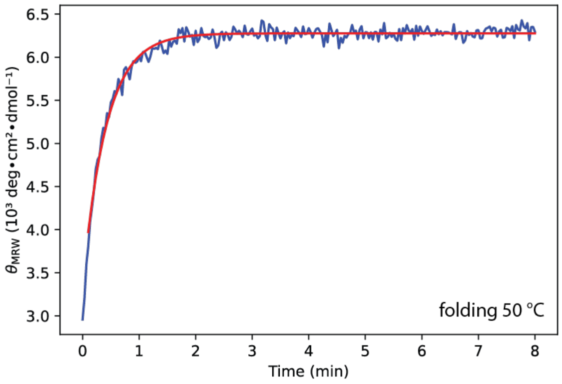

folding 50 °C
θ_{MRW} (10^3 deg•cm^2•dmol^{-1})
Time (min)

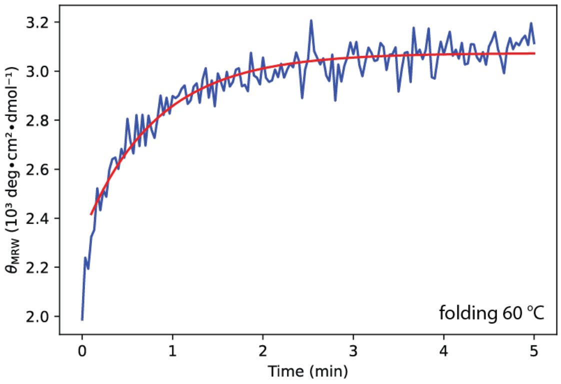

folding 60 °C
θ_{MRW} (10^3 deg•cm^2•dmol^{-1})
Time (min)

Peptide 5

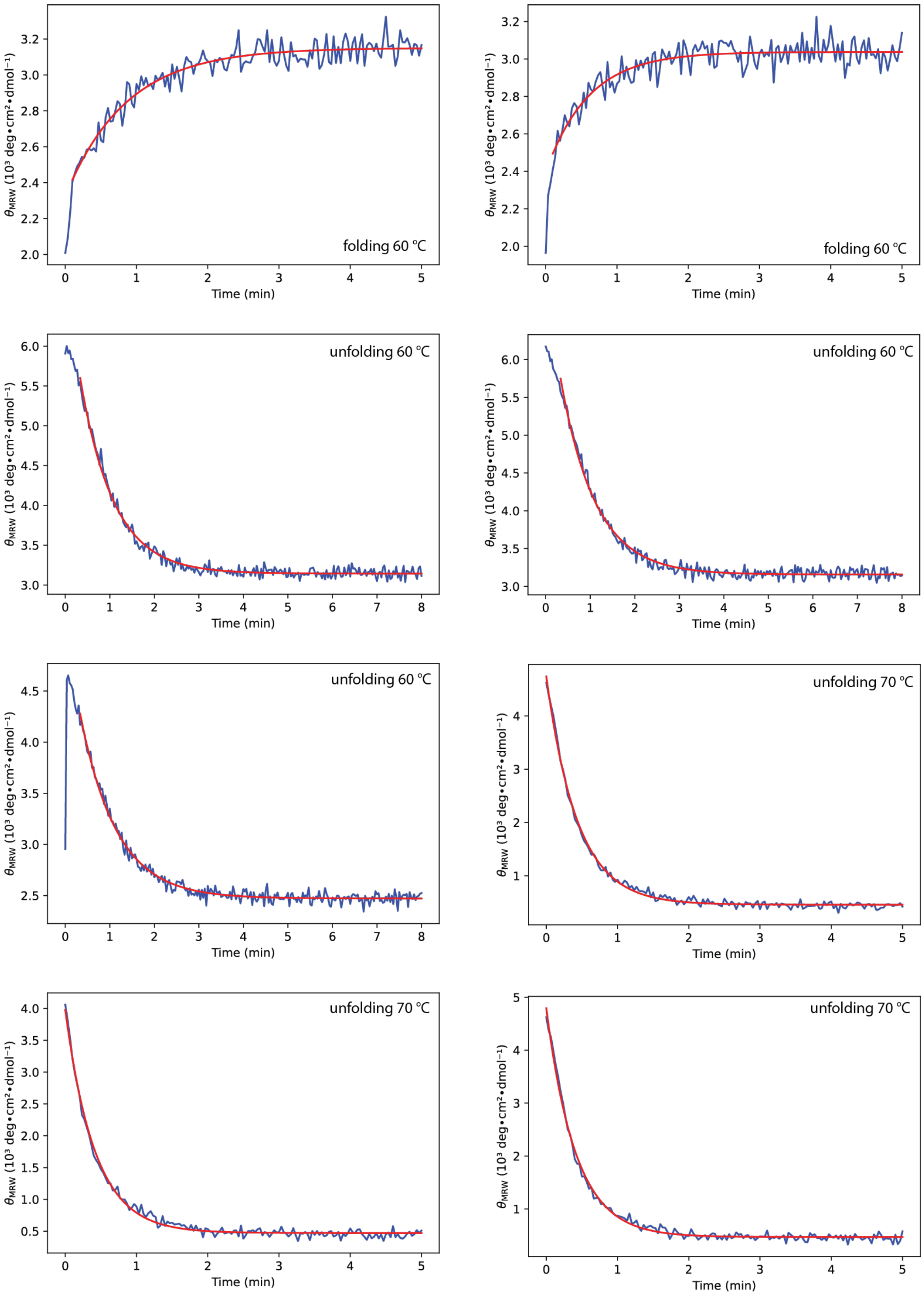

folding 60 ℃
folding 60 ℃
unfolding 60 ℃
unfolding 60 ℃
unfolding 60 ℃
unfolding 70 ℃
unfolding 70 ℃
unfolding 70 ℃
θ_{MRW} (10^3 deg•cm^2•dmol^{-1})
Time (min)

Peptide 5

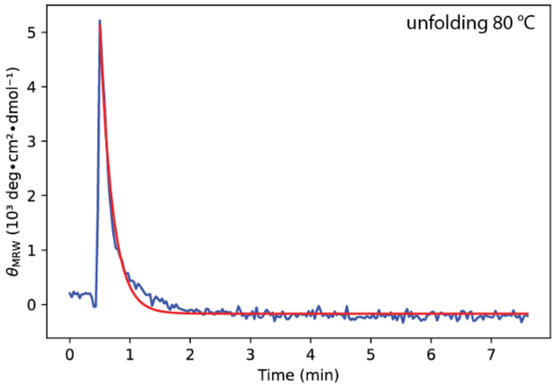
unfolding 80 °C
θ_{MRW} (10³ deg•cm²•dmol⁻¹)
Time (min)

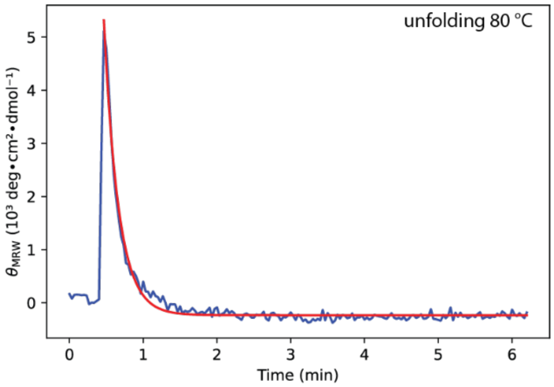
unfolding 80 °C
θ_{MRW} (10³ deg•cm²•dmol⁻¹)
Time (min)

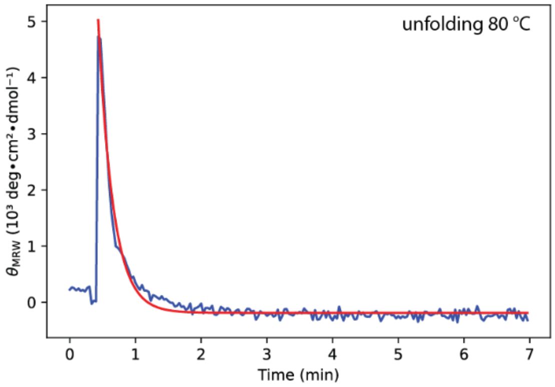
unfolding 80 °C
θ_{MRW} (10³ deg•cm²•dmol⁻¹)
Time (min)

Peptide 6

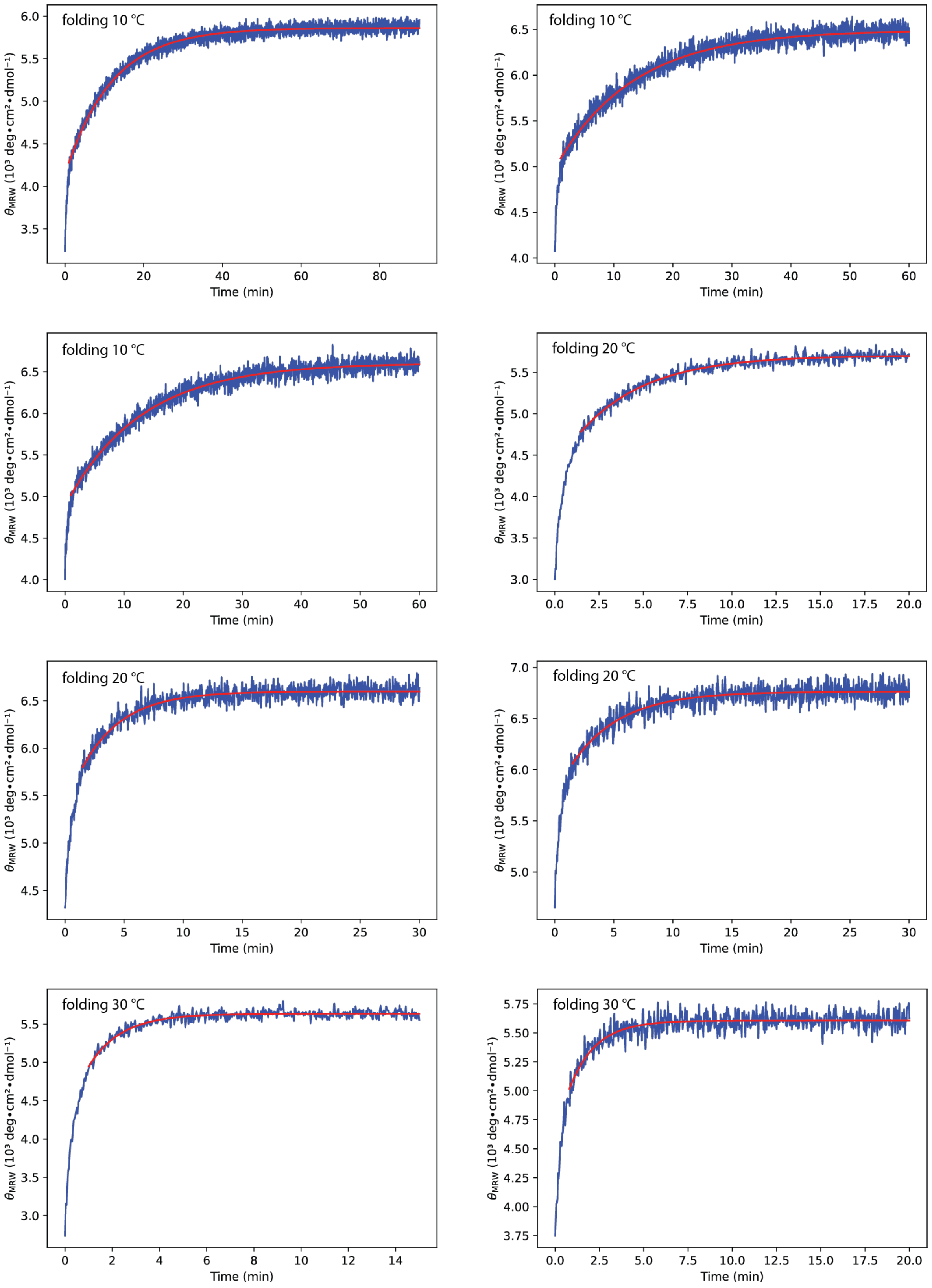
folding 10 °C
folding 10 °C
folding 10 °C
folding 20 °C
folding 20 °C
folding 20 °C
folding 30 °C
folding 30 °C
θ_{MRW} (10³ deg•cm²•dmol⁻¹)
Time (min)

Peptide 6

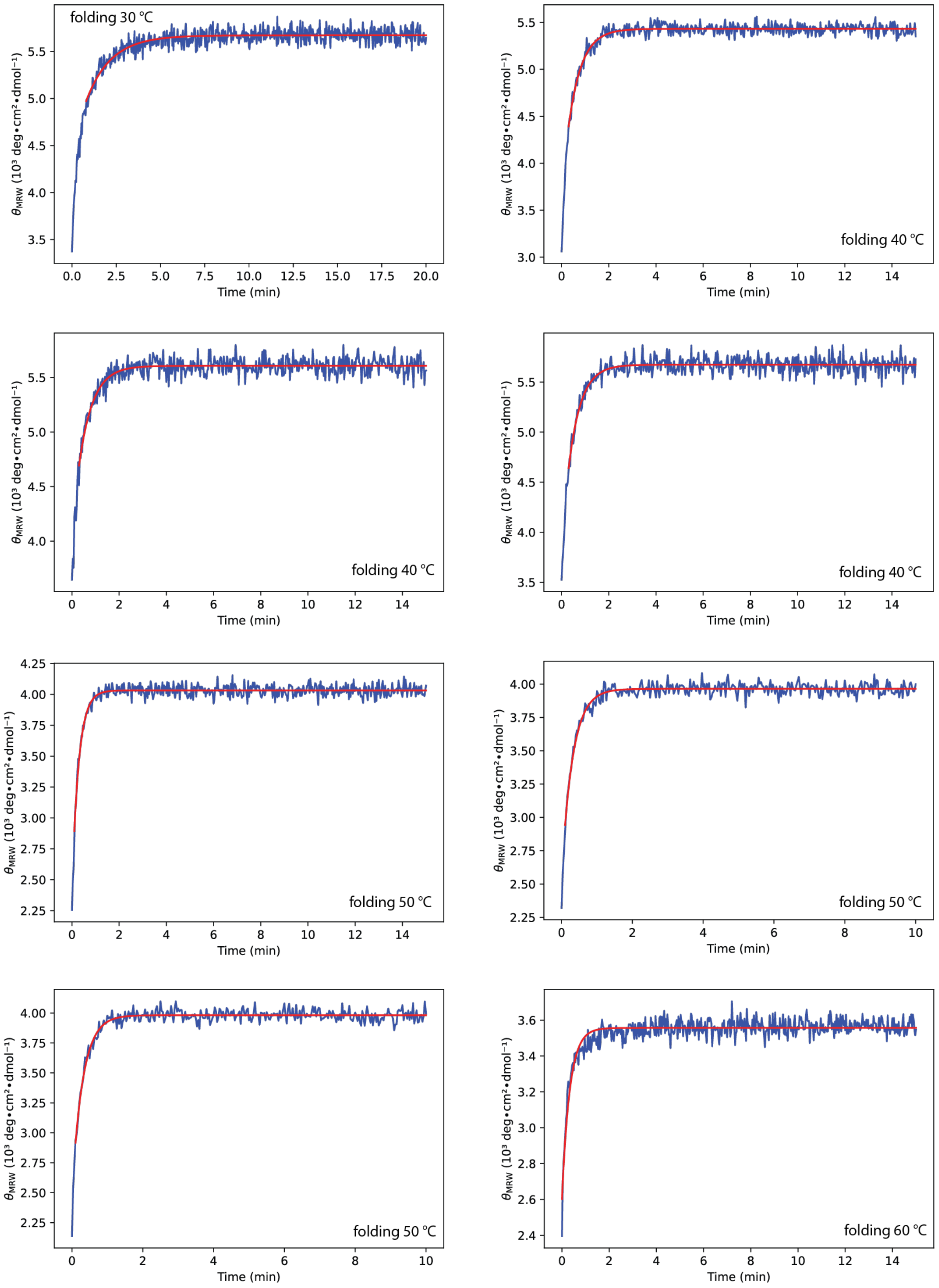
folding 30 °C
folding 40 °C
folding 40 °C
folding 40 °C
folding 50 °C
folding 50 °C
folding 50 °C
folding 60 °C
θ_{MRW} (10³ deg•cm²•dmol⁻¹)
Time (min)

Peptide 6

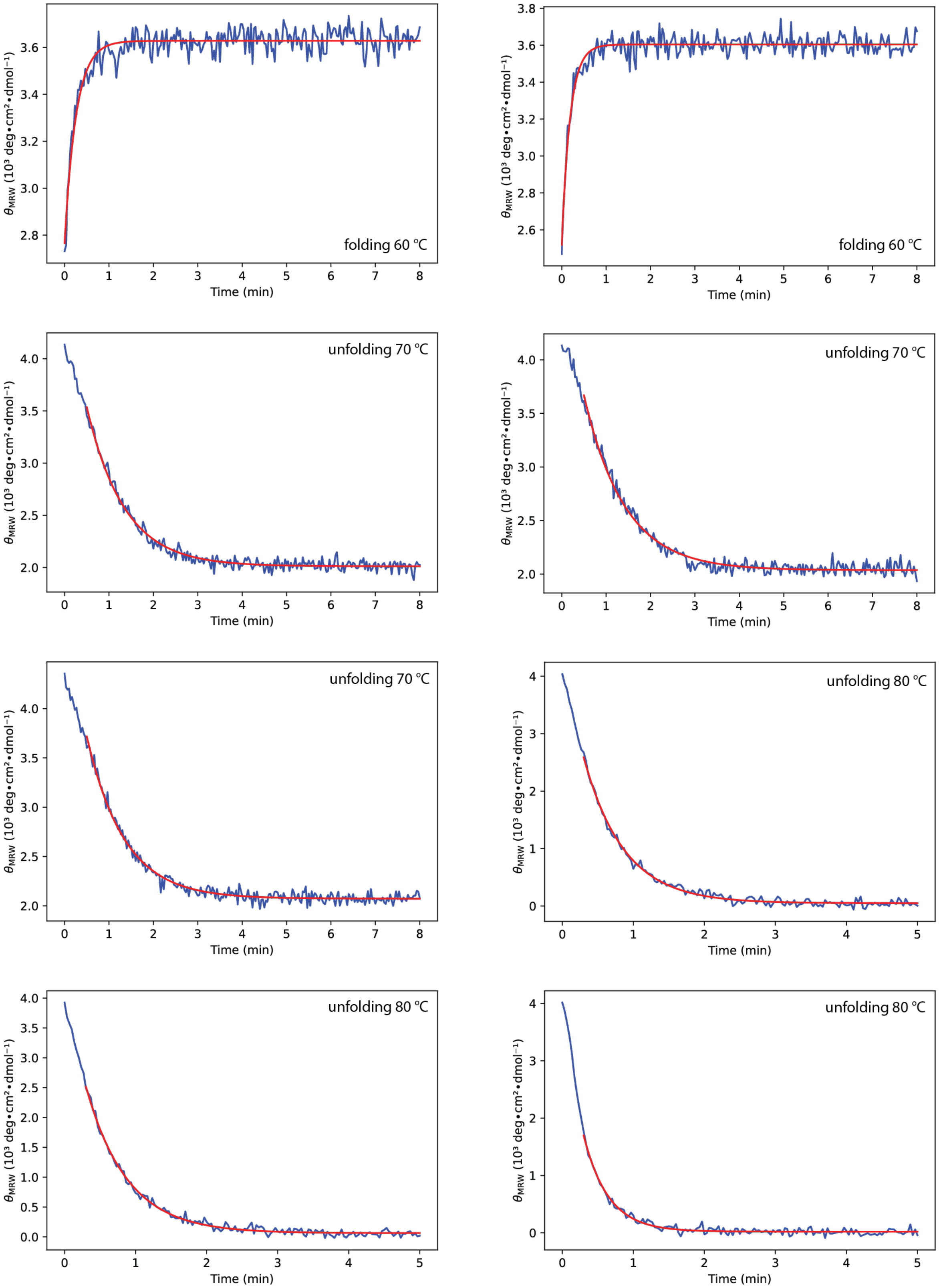
folding 60 °C
folding 60 °C
unfolding 70 °C
unfolding 70 °C
unfolding 70 °C
unfolding 80 °C
unfolding 80 °C
unfolding 80 °C
Time (min)
θ_{MRW} (10³ deg•cm²•dmol⁻¹)

Isothermal Kinetics Measurement
Peptide 4

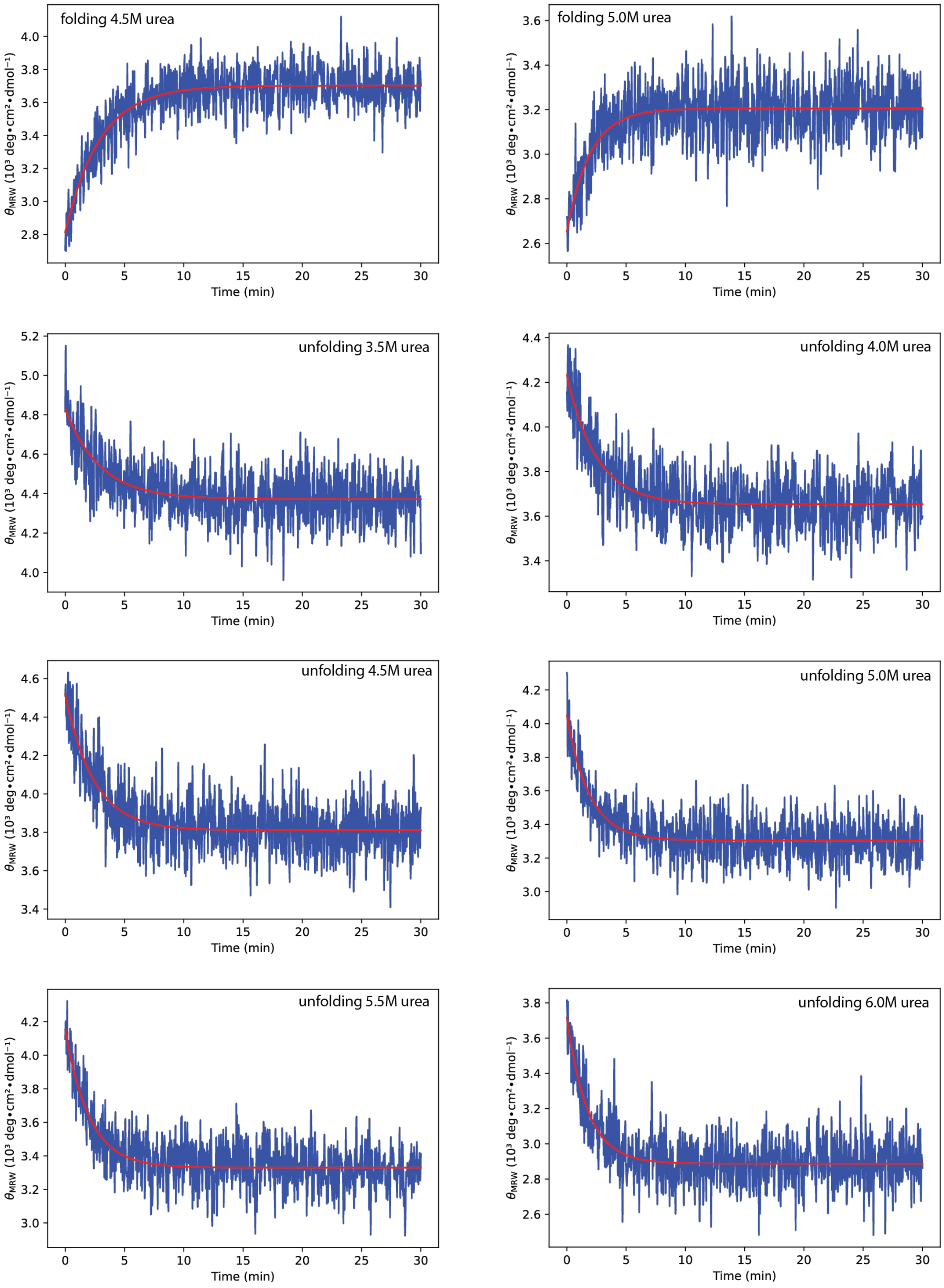

Peptide 4

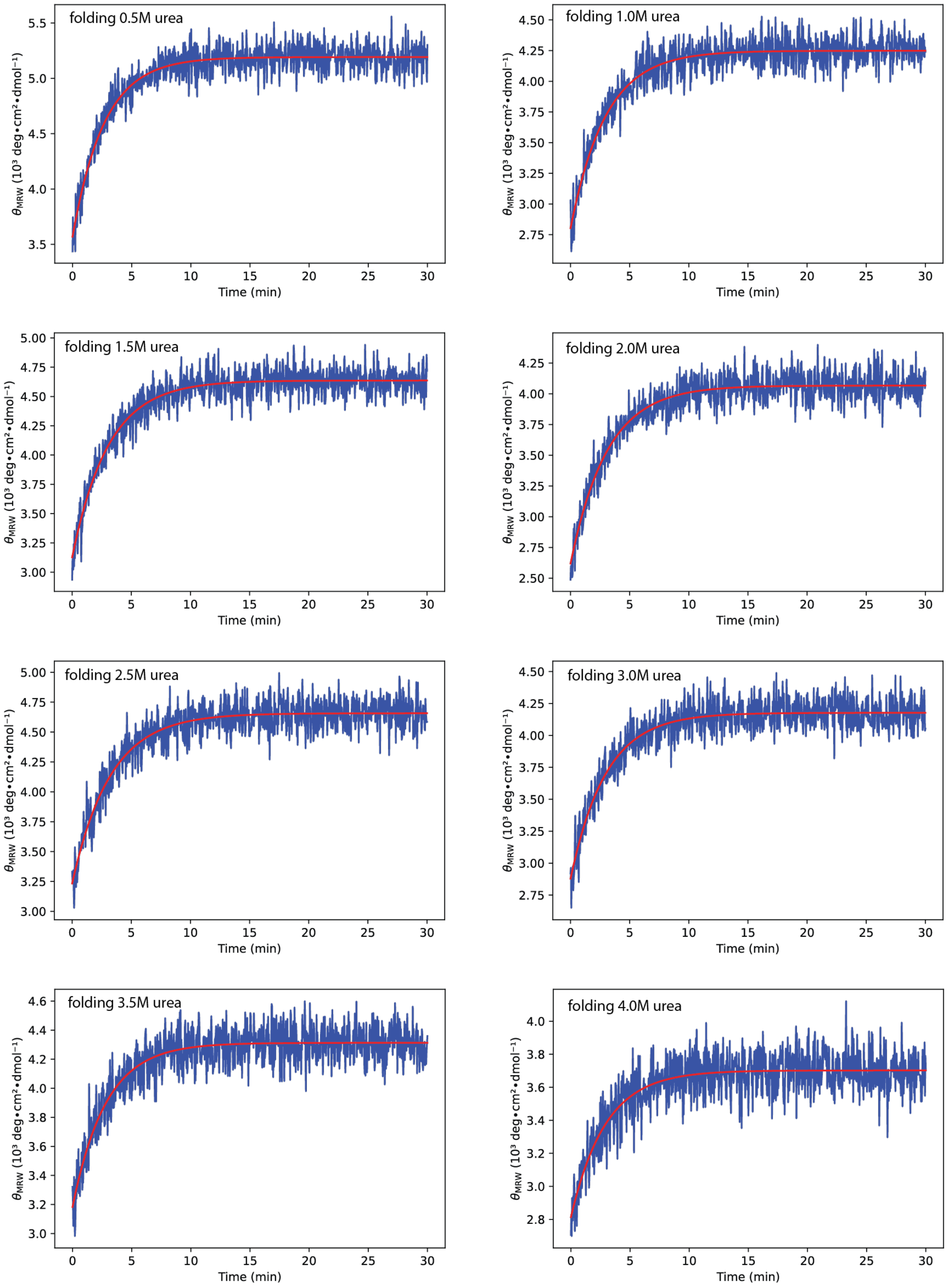
folding 0.5M urea
folding 1.0M urea
folding 1.5M urea
folding 2.0M urea
folding 2.5M urea
folding 3.0M urea
folding 3.5M urea
folding 4.0M urea
θ_{MRW} (10³ deg•cm²•dmol⁻¹)
Time (min)

Peptide 4

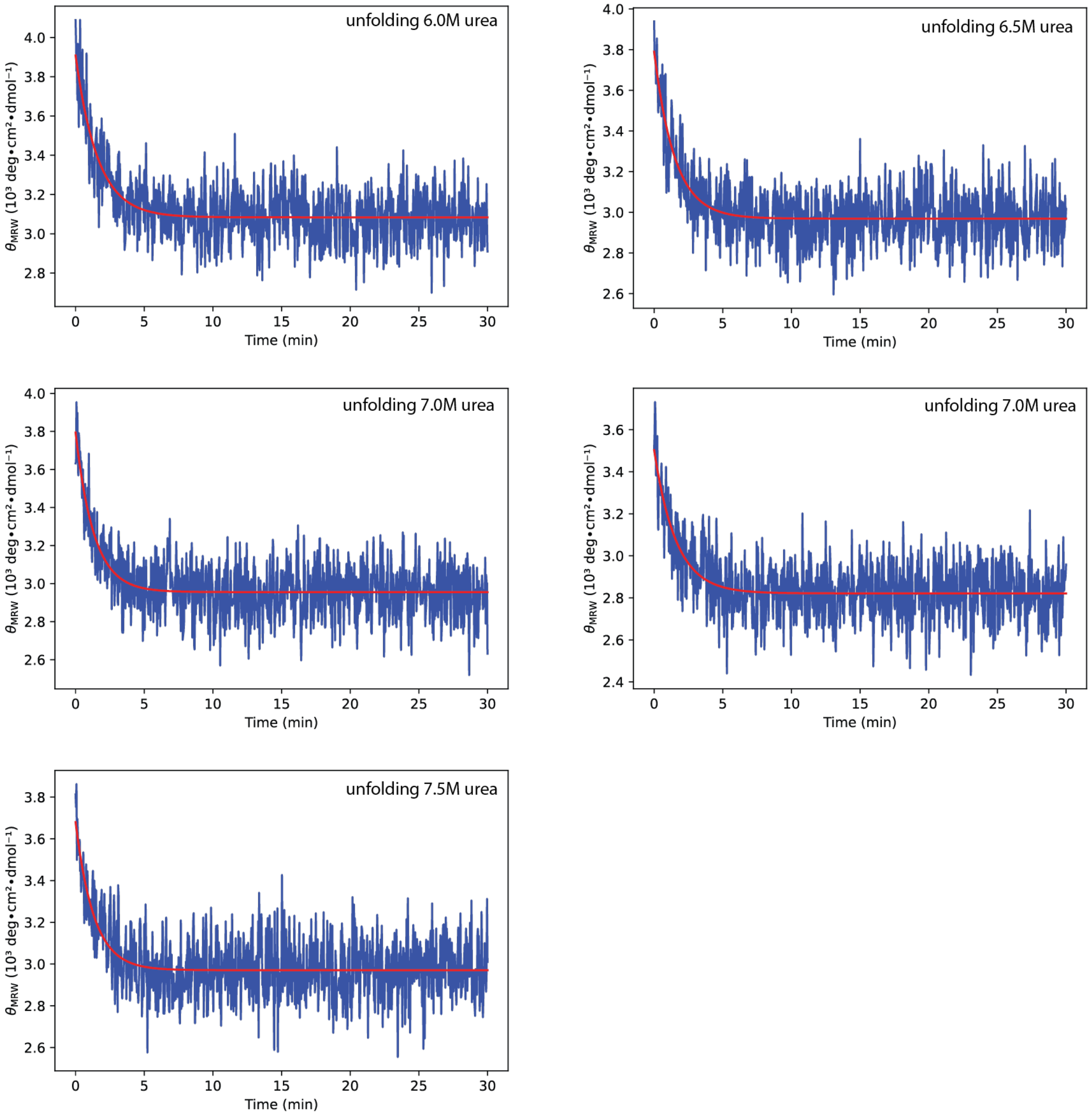
unfolding 6.0M urea
unfolding 6.5M urea
unfolding 7.0M urea
unfolding 7.0M urea
unfolding 7.5M urea
θ_{MRW} (10³ deg•cm²•dmol⁻¹)
Time (min)

References

(1) Zhang, Y.; Malamakal, R. M.; Chenoweth, D. M. Aza-Glycine Induces Collagen Hyperstability. *J Am Chem Soc* **2015**, *137* (39), 12422-12425. DOI: 10.1021/jacs.5b04590.
(2) Zhang, Y.; Herling, M.; Chenoweth, D. M. General Solution for Stabilizing Triple Helical Collagen. *J Am Chem Soc* **2016**, *138* (31), 9751-9754. DOI: 10.1021/jacs.6b03823.
(3) Engel, J.; Chen, H. T.; Prockop, D. J.; Klump, H. The triple helix in equilibrium with coil conversion of collagen-like polytripeptides in aqueous and nonaqueous solvents. Comparison of the thermodynamic parameters and the binding of water to (L-Pro-L-Pro-Gly)n and (L-Pro-L-Hyp-Gly)n. *Biopolymers* **1977**, *16* (3), 601-622, Article. DOI: 10.1002/bip.1977.360160310 Scopus.
(4) Shoulders, M. D.; Satyshur, K. A.; Forest, K. T.; Raines, R. T. Stereoelectronic and steric effects in side chains preorganize a protein main chain. *Proc Natl Acad Sci U S A* **2010**, *107* (2), 559-564, Article. DOI: 10.1073/pnas.0909592107 Scopus.
(5) Virtanen, P.; Gommers, R.; Oliphant, T. E.; Haberland, M.; Reddy, T.; Cournapeau, D.; Burovski, E.; Peterson, P.; Weckesser, W.; Bright, J.; et al. SciPy 1.0: fundamental algorithms for scientific computing in Python. *Nature Methods* **2020**, *17* (3), 261-272. DOI: 10.1038/s41592-019-0686-2.
